# Appraisal terpenoids rich *Boswellia carterri* ethyl acetate extract in binary cyclodextrin oligomer nano complex for improving respiratory distress

**DOI:** 10.1038/s41598-024-66297-2

**Published:** 2024-07-22

**Authors:** Bassant M. M. Ibrahim, Asmaa Badawy Darwish, Sally Abou Taleb, Reda M. Mourad, Noha Nazeeh Yassen, Alyaa F. Hessin, Shaimaa A. Gad, Mona A. Mohammed

**Affiliations:** 1 Pharmacology Department, Medical and Clinical Studies Research Institute, National Research Centre, Dokki, Giza, 12622 Egypt; 2 Pharmaceutical Technology Department, National Research Centre, 33 El-Buhouth Street, Dokki, Giza, 12622 Egypt; 3 Polymers and Pigments Department, Chemical Industries Research Institute, National Research Centre, 33 El-Buhouth Street, Dokki, Giza, 12622 Egypt; 4 Pathology Department, Medical and Clinical Studies Research Institute, National Research Centre, Dokki, Giza, 12622 Egypt; 5 Medicinal and Aromatic Plants Research Department, Pharmaceutical and Drug Industries Research Institute, National Research Centre, Giza, Egypt

**Keywords:** *Boswellia carterri*, Ethyl acetate, Nano sponges, Drug delivery, Sustained release, Respiratory distress, Biochemistry, Plant sciences, Nanoscience and technology

## Abstract

*Boswellia carterii* (BC) resins plants have a long historical background as a treatment for inflammation, as indicated by information originating from multiple countries. Twenty-seven diterpenoids have been identified in ethyl acetate and total methanol BC, comprising seventeen boscartins of the cembrane-type diterpenoids and ten boscartols of the prenylaromadendrane-type diterpenoids. Moreover, twenty-one known triterpenoids have also been found, encompassing nine tirucallane-type, six ursane-type, four oleanane-type, and two lupane-type. The cembrane-type diterpenoids hold a significant position in pharmaceutical chemistry and related industries due to their captivating biological characteristics and promising pharmacological potentials. Extraction of BC, creation and assessment of nano sponges loaded with either *B. carterii* plant extract or DEX, are the subjects of our current investigation. With the use of ultrasound-assisted synthesis, nano sponges were produced. The entrapment efficiency (EE%) of medications in nano sponges was examined using spectrophotometry. Nano sponges were characterized using a number of methods. Within nano sponges, the EE% of medicines varied between 98.52 ± 0.07 and 99.64 ± 1.40%. The nano sponges' particle sizes varied from 105.9 ± 15.9 to 166.8 ± 26.3 nm. Drugs released from nano sponges using the Korsmeyer-Peppas concept. In respiratory distressed rats, the effects of BC plant extract, DEX salt and their nano formulations (D1, D5, P1 and P1), were tested. Treatment significantly reduced ICAM-1, LTB4, and IL*β* 4 levels and improved histopathologic profiles, when compared to the positive control group. *Boswellia* extract and its nano sponge formulation P1 showed promising therapeutic effects. The effect of P1 may be due to synergism between both the extract and the formulation. This effect was achieved by blocking both ICAM-1 and LTB4 pathways, therefore counteracting the effects of talc powder.

## Introduction

*Boswellia carterii*, a member of the Burseraceae family, is widely distributed in Somalia and Ethiopia. Its bark contains secretory tissue that exudes a gum resin. This gum resin, known as olibanum, has been utilized as a medicinal substance in Unani (Islamic) and Chinese traditions for the treatment of various ailments including rheumatoid arthritis, osteoarthritis, dysmenorrhea, ulcers, swelling, and pain resulting from injuries^1^. Previous studies examining the phytochemical and pharmacological properties of olibanum have demonstrated its diverse biological activities, such as anti-inflammatory effects^2^, cytotoxic properties^3^, neuroprotective properties^4^, α-Glucosidase inhibition^5^, and antioxidant activity. Traditional Chinese medicine (TCM) has also employed olibanum to alleviate symptoms associated with traumatic injuries, chest congestion, pain^6^, and inflammatory diseases like rheumatoid arthritis. The resin of BC is widely recognized as a rich source of structurally diverse diterpenoids, with the major classes being triterpenes (such as boswellic acids), oleanane-type compounds, cembrane-type compounds, ursane-type compounds, tirucallane-type compounds, and oxygenated macrocyclic diterpenoid-type compounds. Additionally, prenylaromadendrane-type diterpenes have also been identified in this resin^7^.

Materials that are inhaled in the form of powder target the lungs, an inhaled pneumo-toxicant cause’s serious pulmonary damage via lipid peroxidation and even DNA breakdown^8,9^. Workers subjected to chronic talc powder inhalation are more susibtiple to silicosis and asbestosis, manifested by continuous cough and progressing dyspnoea. If the condition is left untreated, it will progress to pulmonary hypertension and fibrosis^10^. Pulmonary talcosis occurs as a complication of inspiration of large amounts of talc powder^5^. Lung injuries as pneumonia, pleurisy, fibrosis, and haemorrhage were evidenced in rat’s lungs of rats that were subjected to talc powder inhalation. Addiction of talc-adulterated marijuana and prolonged inspiration of talc powder by quarry laborers, lead to talc pneumoconiosis, bronchiolar obstruction and bronchiolitis, and may be fatal^11^.

One kind of corticosteroid medicines that has been widely used as an anti-asthmatic agent is DEX^12,13^. DEX is an immunosuppressant with anti-inflammatory properties that can relieve the inflammation and pathophysiology of asthma^14^. Regrettably, the administration of oral corticosteroids over an extended period of time has been marred by the occurrence of systemic adverse reactions, such as renal insufficiency, hypotension, reduced body mass, conjunctival inflammation, and visual impairment, notwithstanding the drug's profound curative potency^15^. In view of this, there is a growing necessity to formulate a strategy to optimize the curative capabilities of corticosteroids while alleviating their systemic adverse effects^16^.

The utilization of a pulmonary route for drug delivery is extremely attractive and has gained significant attention for the treatment of respiratory diseases and conditions. This is primarily due to the lung's expansive surface area, its rich blood supply, and its ability to effectively absorb medications for both local and systemic distribution^17–19^. In comparison to more conventional methods of drug delivery such as oral, peritoneal, and systemic administration, pulmonary medication delivery offers distinct advantages. By directly targeting the airway, this form of administration bypasses the first pass effect and allows for the precise treatment of pulmonary conditions. Consequently, medications delivered via the pulmonary route can elicit therapeutic effects at lower concentrations compared to alternative routes, thereby minimizing systemic side effects. However, it has been argued that a significant portion of inhaled corticosteroids remains within the oral cavity and permeates into the surrounding tissue, resulting in adverse effects^19^. In order to overcome these challenges in pulmonary medication delivery, the use of drug carriers that effectively transport pharmaceuticals to the desired site while maintaining appropriate drug concentrations within the airway is of utmost importance^20^.

A considerable amount of effort has been devoted to the design of colloidal drug delivery systems in order to enhance the therapeutic effectiveness of drugs by modifying their bio-distribution and pharmacokinetics^13^. Liposomes, solid lipid nanoparticles, and polymeric nanoparticles have all undergone extensive investigation for drug delivery in the lungs. One of the innovative drug carriers that have recorded an enhanced effect is nano sponges. Chemically crosslinked polymers known as nano sponges are produced by reacting the CD unit with an appropriate crosslinking agent, such as CA, BDE, PMDA, CDI, or DPC^21^. *β* CD-NS has an amazing ability to encapsulate. They create novel drug carriers, safeguard biodegradable materials, enhance the aqueous solubility of weakly water-soluble compounds, or provide long-lasting delivery systems. Parenteral, pulmonary, and oral routes can all be impacted by the spherical form and tiny size of NS^22,23^.

The goal of this work is to prepare and evaluate ethyl acetate extract from *Boswellia carterri* and then nano sponge formulations loaded with DEX salt compared to *Boswellia carterri* extract nano sponge for pulmonary administration intended for the treatment of respiratory allergies and asthma to enhance their efficacy, avoiding hepatic first-pass metabolism as well as the severe side effects that accompany orally administered drugs such as gastric irritation, and, moreover, to overcome compliance problems, thus providing better convenience of treatment.

## Materials and methods

### Materials

BC resins were gathered from the governorate of Khartoum in Sudan, where this collection was conducted in accordance with both local and national guidelines, as proper permission had been obtained for the collection of plant material. Dexamethasone Sodium Phosphate was graciously provided as a gift from the esteemed AMRIYA Pharmaceutical Industry Company in Egypt. Hydroxypropyl *β*-cyclodextrin (HP*β*-CD) (KLEPTOSE HPB, MW 1380) and epichlorohydrin-*β*-cyclodextrin (EPI-*β*-CD) were kindly supplied by the reputable company Roquette in France. Diphenyl carbonate (DPC) was procured from the esteemed establishment Acros Organics in Belgium. Prednisolone was obtained from the well-known Sigma Chemical Co. (St Louis, MO, U.S.A.). Disodium hydrogen phosphate anhydrous and Potassium dihydrogen phosphate were purchased from El-Gomhouria Pharmaceutical Chemicals in Egypt. The cellulose membrane was acquired from the esteemed company Sigma-Aldrich, chemie Gmbh, located in Steinheim, Germany. All other chemicals and solvents used are of chemical grade and were utilized without any additional purification.

Talc powder for induction of respiratory distress, was purchased from local medical suppliers; brand name “Five Fives Baby Mary Talcum”, it is high quality fragrance and clumps free powder, each powder pack weighs 200 gm**.** Diethyl ether and Formaldehyde were purchased from “Sigma Chemical Co., St. Louis, MO, USA”, the former for induction of anaesthesia before withdrawal of blood and the later for fixation of postmortem tissues. Diagnostic Elisa kits: “Intracellular adhesion molecule 1(ICAM-1-1), Leukotriene B4 (LTB 4), Interleukin*β* 4 (IL*β* 4)”, for assessment of allergic and inflammatory markers levels in serum were purchased from “Elabscience Inc (USA)”, their measurement procedures followed the manufacturer’s guidelines. Trolox from Sigma Aldrich, all other chemicals and solvents were of high analytical grade.

*Animals* In the present study, female Wister albino rats were used. Fifty animals of body weights (150–175 g) were obtained from the animal house colony of National research center in Egypt. They were housed in metal cages of uniform weights and designs, in well aeriated room at temperature range 22 ± 3 °C, and humidity range 55 ± 5%. Standard chow and free water access were available. The study was done in accordance with the guide for care and use of laboratory animals Approval of the ethics committee of “National Research Centre” numbered 19/209 was obtained prior to performance of the study^24^. The experiments were consistent with the civil regulations of “Animal Welfare and the Institutional Animal Ethical Committee (IAEC)”, and were congruent with the “Animal Research: Reporting of in vivo Experiments (ARRIVE)” guidelines^25^.

### Methods

#### Phytochemisty section

##### Plant materials and extraction

*Boswellia carterii* was acquired from the city of Khartoum, located in the Republic of Sudan. It was identified by *Dr. Mohamed El Gebally*, Former researcher of plant taxonomy, National Research Center and Senior of plant taxonomy at Orman Botanical garden, Giza, Egypt^24^. Herbarium specimen number M227 was deposited at the herbarium of the National Research Centre, Dokki, Cairo, Egypt^26^. The desiccated resin, weighing 500 g, underwent a comprehensive extraction process using 3 L of methanol at ambient temperature, repeated thrice. Subsequently, the extract was subjected to filtration and concentrated under reduced pressure at a temperature of 45 °C, employing a rotary evaporator^27^. The resulting unrefined residue was immersed in water, allowing for an overnight duration, and underwent a sequential partitioning procedure involving chloroform, followed by ethyl acetate, n-butanol, and ultimately, water^28^.

##### Phytochemicals screening

BC extracts were conducted to identify the presence of various compounds. The methanolic, dichloromethane, ethyl acetate, butanol and water extracts were examined for the presence of carbohydrates and/or glycosides using the a-naphthol sulphuric acid reagent, as previously described by Lewis and Smith^29^. Tannins were detected using the method developed by Shellard^30^. To test for alkaloids, one mL of the alcoholic extract filtrate was mixed with 2 mL of Dragendoff's reagent^31^. The presence of alkaloids was indicated by the formation of a turbid orange color. Mayer's reagent was used as a confirmation test for alkaloids, and the appearance of a yellow precipitate confirmed their presence^32^. The potential presence of flavonoids was determined by the formation of a yellow color according to Trease and Evans^33^. Additionally, the extracts were treated with magnesium / HCl, and the formation of a red color indicated the possible presence of flavanones and/or flavonol^34^. Saponins were identified if a froth persisted for approximately 30 min^30^. Lastly, a green coloration in the upper layer and a deep red color in the lower layer indicated the presence of steroids and triterpenoids, respectively, as reported by Hanson^35^.

##### Metabolomics of secondary metabolites from BC resins using LC/MS/MS

10 mg of two extract were dissolved in 1 ml of 80% concentration methanol and then filtrated by a sarangi filter. The diluted extract was repeated three times.

The UPLC system (specifically the Acquity system from Waters, located in Milford, USA) was connected to the Q-Exactive hybrid MS/MS quadrupole—Orbitrap mass spectrometer (manufactured by Thermo in Germany). To achieve chromatographic separation in this system, a water solution acidified with 0.1% formic acid (referred to as solvent A), and acetonitrile (referred to as solvent B), were used with a mobile phase flow rate of 0.3 mL/min. The gradient for the separation process was as follows: from 0 to 7 min the composition changed from 50% solvent A to 50% solvent B, and from 7 to 15 min it transitioned to 98% solvent B. These conditions were maintained for a total of 17 min. The separation was performed using the BEH shield C18 column, which had dimensions of 150 × 2.1 mm and 1.7 μm particle sizes. The Q-Exactive MS was operated with the following settings done by Piasecka et al.^36^.

##### Processing data

We have developed a methodology for determining the fragmentation patterns of 49 metabolites using mass spectrometry. This comprehensive approach includes information on retention time and MS/MS data. The raw MS data for the two fractions (methanol and ethyl acetate) of BC metabolites were exported in a standardized output format (abf) and analyzed using MS-DIAL 4.18, a software tool that offers improved and standardized untargeted metabolomics data analysis. To eliminate noisy spectra, the MSP format was employed along with a classical spectral similarity calculation. The potential metabolites of interest were identified by comparing their fragmentation patterns and RI with those found in the REAXYS, KNAPSACK, and RIKEN databases^37,38^.

#### Nano-section

##### Preparation of nano sponges

A modified version of the ultrasound-assisted synthesis approach was employed to synthesize NSs. Diphenyl carbonate, employed as a cross-linker, and HP*β*-CD, utilized as a polymer, was combined in distilled water at a predetermined molar ratio^39,40^. Table 4 shows the ratios of HP*β*-CD: DPC that were employed (1:5, 3:1, 4:1, and 5:1). At 90 °C, the mixture was sonicated, and then homogenized in a hot water bath for seven minutes at 12,000 rpm. After being moved to falcon tubes, the mixture was centrifuged for 30 min at 6000 rpm. Following centrifugation, the drug was added, and the mixture was shaken at 150 rpm for the whole night. The mixture was centrifuged at 6000 for 30 min at room temperature after being sonicated at 90 °C the next day. After transferring the mixture to a petri dish and adjusting the volume for freeze drying, it was kept at 25 °C until needed again. This process will yield spherical, uniformly sized nano sponges.

##### Determination of encapsulation efficiency (EE %)

The samples underwent filtration by means of a 0.22 µm membrane filter and were subsequently subjected to analysis at the predetermined λ_max_, specifically at 242 nm^41^, utilizing an ultraviolet–visible (UV) spectrophotometer (Pharma spec 1700, Shimadzu, Japan). The estimation of encapsulation efficiency for all ratios was conducted through the utilization of the subsequent equation: EE% = *(M*_*act*_*/M*_*the*_*)* × 100.where M_act_ = actual DEX content in weighed quantity of nano sponges, and M_the_ = theoretical DEX content in nano sponges^42^.

##### Vesicle size, polydispersity index and zeta potential measurement

The samples distributed in double-distilled water were subjected to examination of PS, ZP, and PDI using Zeta-sizer (Nano Series ZS90, Malvern Instruments Ltd., Worcestershire, UK) and dynamic light scattering (DLS)^43^.

##### Preparation of the optimized formulations

Following the completion of earlier trials, the ideal molar ratio for generating DEX salt NSs with the best EE%, PS, and ZP values was chosen to be the optimum formulation. Then, BC plant extract NSs (P1) were prepared using the chosen molar ratio of HP*β*-CD: DPC. In the meantime, a novel complex containing HPB-CD and epichlorohydrin-*β* -cyclodextrin (EPI-*β* -CD) was created at the chosen molar ratio, and it was loaded with plant extract (P2) and Dex salt (D5) as shown in Table 4. The new formulations were prepared using the same procedure that was described in Sect. “Determination of EE%, PS, ZP and PDI of the optimized formulations”.

##### Determination of EE%, PS, ZP and PDI of the optimized formulations

As stated in Sect. (“Characterization of the optimized formulations”), the resulting DEX salt and plant formulation’s EE% was measured at predetermined λ_max_ 242 and 252 nm respectively. Table 4 displays the composition, encapsulation efficiency, and physico-chemical parameters of the optimized formulations.

##### Characterization of the optimized formulations

*Surface morphology* Transmission electron microscopy (TEM): The selected formulations' morphological features were analyzed through the use of TEM (JEOL Co., JEM-2100, Japan). After applying one drop of the diluted sample to a carbon-coated copper grid, the samples were stained after the grid had dried for fifteen minutes at room temperature. The grid was sprayed with a drop of 1%w/v phosphotungstic acid solution, allowed to stand for three minutes, and then placed under the microscope to examine the samples at the appropriate magnifications for surface characteristics and shape.

Scanning electron microscopy (SEM): Using a scanning electron microscope (SEM) (Quanta FEG 250, ThermoFisher Scientific Co., Czech Republic), the surfaces of the chosen formulations were analyzed. Using double-sided tape, freeze-dried samples were attached to aluminum stubs and then coated with a thin layer of gold using a sputter coater unit. The SEM was run at a distance of 10 mm and an acceleration voltage of 20 kV.

*Fourier transform infrared spectroscopy (FTIR) analysis* FT-IR analysis was used to identify any potential interactions between the blend of chosen formulations and the Nano sponge’s components. The infrared spectrum (400–4000 cm^–1^) was used to scan the samples (JASCO 6100, Tokyo, Japan). Potassium bromide pellets were used to prepare the FT-IR samples.

##### In-vitro release study

In this investigation, release study of DEX and Plant NSs formulations (D1, D5, P1 and P2) as well as free DEX and Plant solutions was performed. The dialysis bags, specifically made of Dialysis tubing cellulose membrane obtained from Sigma-Aldrich Co., located in St. Louis, USA, with a molecular weight cut-off ranging from 12,000 to 14,000, were appropriately filled with a quantity equivalent to 2 mg of the NSs formulations alongside aqueous solutions of DEX and plant extract. Prior to being suspended in screw-capped glass containers with a capacity of 100 ml, which were also filled with 100 ml of PBS with a pH value of 7.4 in order to maintain sink condition, the dialysis bags were meticulously sealed on both ends to effectively prevent any leakage from occurring. It is worth noting that this procedure was done to ensure the integrity and accuracy of the results^44,45^. The entire experiment took place within a controlled environment, namely a thermostatic shaking water bath provided by Memmert, model SV 1422, which is a reputable brand based in Germany. The temperature was set at a constant 37 °C with a tolerance of 0.5 °C, while the shaking speed was maintained at 100 revolutions per minute. Throughout the course of the experiment, samples were regularly collected at specific time intervals and simultaneously replaced with an equal volume of the designated replacement release medium. This meticulous process was carried out to sustain the desired sink state and ensure the reliability of the findings. In order to compare the DEX and plant concentrations in the removed samples to blanks that had received the same treatment, spectrophotometric analysis was used. By dividing the amount of drug released by the amount of drug in the dialysis bag at the beginning, the cumulative release percentages were calculated. Three distinct samples were used for each measurement, which was done in triplicate.

Numerous mathematical models, including Higuchi's square root of time model, zero, first order and Korsmeyer–Peppas kinetic models, were used in the kinetic investigation of drug release from NSs formulations^46,47^. The plots of Q vs. t in the case of zero order, log (Q0–Q) vs. t for first order, and Q vs. t1/2 for the Higuchi model were used to derive the R2 values that indicate the coefficient of determination. Where (Q) is the amount of drug that has been released at time (t) and (Q0–Q) is the amount of drug that is still present at time (t). The release exponent (n) describing the mechanism of drug release from the matrices was calculated by regression analysis using the following equation:

M^t^/M^∞^ = kt^n^.

where Mt/M∞ is the fraction of drug released at time t and k is a constant incorporating the structural and geometric characteristics of the release device. When n = 0.5, case I or Fickian diffusion is indicated, 0.5 < n < 1 for anomalous (non-Fickian) diffusion, n = 1 for case II transport (zero-order release), and n > 1 indicates super case II transport^48^. The most accurate model was considered to have the highest correlation coefficient values or determination coefficient (R2).

#### Pharmacological section

##### In vitro study of DPPH and ABTS antioxidant activity

The method employed to evaluate the in vitro antioxidant activity of two BC plant extracts was the DPPH and ABTS free radical-scavenging activity method. Different concentrations (30, 20, 10, 5, 2.5, 1.5, 1 and 0.5 μg/mL) were utilized for this purpose. As a positive control, ascorbic acid and trolox were employed^49^.

The estimation of ABTS^+^ in various extracts was conducted using the methodologies outlined by Dinkova-Kostova et al.^50^. The DPPH (1,1-diphenyl-2-picrylhydrazyl, 250 mM) radical scavenging assay was carried out as described by Mohammed et al.^51^. To calculate the percentage inhibition of the DPPH and ABTS^+^ radicals, the following formula was utilized: % inhibition = [(A_control_–A_sample_)/A_control_] × 100. In the case of DPPH, A represents the absorbance at 517 nm, while for ABTS + , A is the absorbance at 734 nm^52^.

##### In vivo pharmacological study

*Experimental design of the efficacy study* Fifty female Wistar albino rats were used for evaluation of the efficacies of the *Boswellia carterii* extract, DEX salt and the tested formulations. The rats were divided into 10 equal groups (n = 5). Rats in group I acted as the negative control group and received oral saline solution 0.9%. The other nine groups were exposed to 50 mg/m^3^ talc for 6 h daily, 5 days/week for 4 weeks for induction of respiratory distress^53^. After 4 weeks of exposure to talc powder, they were subdivided into the following groups: group II which was untreated and served as positive control group, and treated groups III, IV, V, VI, VII, VIII, IX and X which received Dexa salt, D1, D5, *Boswellia carterii* plant extract, P1, P2*,* Drug-free NSs DF1, Drug-free NSs DF2, respectively. All were given in doses of 10 µl which was equivalent to 20 µg of each substance. All were instilled in each nostril (IN) for 4 weeks following cessation of exposure to talc powder.

Each rat was examined daily pre and post exposure to talc powder and during treatment to observe any symptoms of respiratory distress such as either apnoea or dyspnoea manifested by cyanosis around mouth, panting or lethargy. After the terminal treatment dose, the rats were fasted for about 16 h before blood sampling for biochemical assay and dissection for histopathological examination^54^.

*Blood sampling and biochemical estimation of anti-inflammatory and anti-allergic biomarkers* Sixteen hours after last dose of treatment, all rats were anaesthetized and the “retro-orbital plexus of veins” was punctured for blood sampling. Then the samples were placed in the centrifuge and the rotating speed was set at 1500 rpm for duration of 10 min in order to get clear serum.

Biochemical analysis was done by using “ELISA kits” following manufacturer’s guidelines for rat “ICAM-1-1, IL*β* 4 or leukotriene B4”.

*ELISA tests principles:* The ELISA kit employed the principle of "Sandwich-ELISA". The kits were equipped with micro ELISA plates that had already been coated with an antibody that specifically targeted either Rat ICAM-1, IL*β* -4, or leukotriene B4. The micro ELISA plate wells were then subjected to the addition of standards or samples, which were subsequently combined with the specific antibody. Following this, a biotinylated detection antibody that specifically targeted rat ICAM-1-1, IL*β* 4, or leukotriene B4, as well as Avidin-Horseradish Peroxidase (HRP) conjugate, were added successively to each micro plate well and incubated. The removal of free components was achieved through a thorough washing process. Subsequently, the addition of the substrate solution to each well took place. It was observed that only the wells containing rat ICAM-1–1, IL*β* 4, or LTB4 displayed a blue coloration resulting from the presence of the biotinylated detection antibody and Avidin-HRP conjugate. The enzyme–substrate reaction was brought to a halt by the addition of the stop solution, leading to a yellow coloration.

- The elisa plate reader was employed to measure the optical density (OD) at a wavelength of 450 nm ± 2 nm. It was established that the OD value was directly proportional to the concentration of either Rat ICAM-1-1, IL*β* 4, or LT B4.
- The concentrations of rat serum ICAM-1-1, IL*β* 4, or LTB4 were determined by comparing the OD of the samples to the standard curve.

*Tissue preparation for histopathological examination* All animals were sacrificed by euthanasia. All parts of the upper and lower respiratory tract from each animal were excised and fixed in 10% neutral formalin solution for 24 h, followed by washing with tap water, dehydrating in alcohol, clearing in xylene and ultimately embedding in paraffin^55^. Serial sections of 3 µm thickness were cut followed by staining with hematoxylin and eosin^51^, for preparation for histopathological examination*.* All images were captured at “The Pathology Lab in the National research Centre in Egypt” by using the image analysis system with a light microscope “Olympus CX41” and “SC100 video camera” that were attached to a computer system. All the photomicrographs that were taken at different magnifications were processed using “Adobe Photoshop version 8.0”^56^.

##### Statistical analysis

All values were expressed as means of the results of biochemical parameters plus or minus the standard error. Comparisons between all means were conducted using the One Way Analysis of Variance (ANOVA) followed by the Tukey Kramer's test for multiple comparisons. A significance level of P ≤ 0.001 was considered statistically significant. The eighth version of the Graph pad prism software was utilized to perform all the statistical tests^57^.

## Results and discussions

### Phytochemical screening of BC and their fractions

After defatted resin powder, 100% methanol (2L) was added and kept overnight on shaking 120 rpm. The solvent was evaporated by rotary evaporator at 40 °C till dryness. The crude residue was suspended in water, left for a duration of 24 h, and subsequently divided into four portions using 0.8ml of chloroform, followed by 0.8ml of ethyl acetate, 0.8ml of n-butanol, and finally 0.8ml of water.

The findings demonstrated the existence of flavonoids, carbohydrates, tannins, triterpenoids, steroids, alkaloids, and saponin. These substances were displayed in (Table 1). Flavonoids and saponin were detected in the ethyl acetate, butanol, and water extracts. These findings align with the outcomes reported by Ref.^58^, who identified the presence of flavonoids, carbohydrates, saponins, tannins, phenol, coumarins, and triterpenes. However, carbohydrates and tannins were not present in the chloroform extract (Table 1)^39^.

**Table 1:** Phytochemical screening of *Boswellia carterii* and their fractions.

Groups	*Boswellia carterii resins*				
	Total	CH_2_CL_2_	EtOAc	n-Butanol	H_2_O
Volatile oils	+ +	–	–	–	
Carbohydrate	+ +	–	+ + +	+ + +	+ +
Tannins	+ +	–	+ + +	+ + +	+
Flavonoids, NaOH	+ +	+ +	+ + +	+ +	+
Flavonoids (Shinoda test)	+ +	+ +	+ + +	+ +	+
Saponin	+ + +	+ +	+ + +	+ + +	+
Sterol and/or triterpenes	+ + +	–	–	–	–
Coumarins	+ + +	+ +	+ +	+	–
Alkaloids	+	+	+	–	–

(+ +), ( +) and (–) refer to high, low and absente amount respectively.

### UPLC-HRMS profiles of *BC-*resins by LC-MSMS

**Figure 1:**
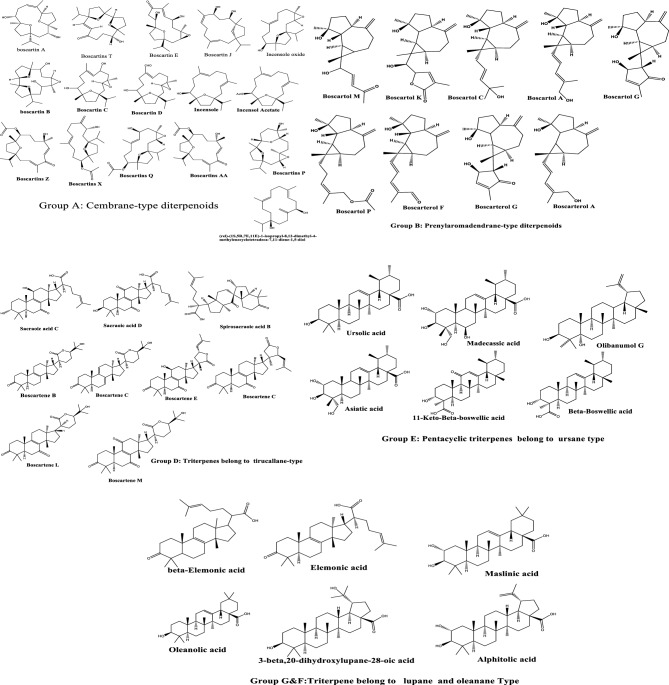
Illustrates the numerical representation of the compounds listed in Table 3. The chemical structure depicted in Figure pertains to various groups, namely Group (**A**–**F**), which encompasses cembrane-type diterpenoids, prenylaromadendrane-type diterpenoids, as well as tirucallane-type, ursane-type, oleanane-type, and lupane-type triterpenes. These compounds were identified in BC through the use of ethylacetate and total methanol extract.

The metabolomics profiles of compounds examined by LC–MS/MS of *Boswellia carterii* (BC) were documented in Table 2. This table includes a total of 49 compounds and is visually illustrated in (Fig. 1) and (Supplementary Figure S1). Cembrane-type diterpenes, which are a diverse group of oxygenated macrocyclic diterpenoids composed of 14-membered rings, represent a prominent class of secondary metabolites predominantly found in the coral genera *Sinularia*,* Sarcophyton*, *Lobophytum*, *Tabacoo*, and *Boswellia*. Twenty-six diterpenoids, classified as sixteen cembrane-type diterpenoids boscartins and ten prenylaromadendrane-type diterpenoids boscartols, along with twenty known triterpenoids, belong to nine tirucallane-types, six ursane-type, three oleanane-type, and two lupane-type. Their structure elucidations were achieved by the LC-MSMS examination. Compounds listed in the table were found in total methanol and ethyl acetate extract; these are compounds compared with the literature, compounds identified from the electronic databases as KNApSAcK databases, and compounds identified from MS_Dial. This paper deals with many of the literature searches listed in (Table 2).

**Table 2:** Metabolomics profiles of listed compounds by LC-MSMS.

No	RT	Compounds names/SMILES	Chemical formula	Mass				∆ppm	PDA	Extracts		Refs.
				Measured & calculated	Exact mass of [M + H]^+^	Measured &calculated	Exact mass of [M-H]-			Total	EtOAc	
Group A: cembrane-type diterpenoids												
3	6.30	Boscartins T O = C(/C([H]) = C1\[H])CC[C@@]([H])(O)[C@](CC2)(C)O[C@]2([H])[C@@]3(C)CC[C@@]1(O3)C(C)C	C_19_H_30_O_4_	323.2212, 323.2217	305.1740[C_18_H_25_O_4_], 287.2006[C_19_H_27_O_2_], 241.1590[C_17_H_21_O]	321.2116, 321.2060	199.1695[C_12_H_29_O_2_]	− 1.6244	245		*	^1^
4	6.59	Boscartin E O[C@H](/C = C1\C)C[C@](O2)(C)[C@@H]2C[C@]3(C(C)C)CC[C@](O3)(C)[C@H](O)CC1 = O	C_20_H_32_O_5_	353.2231, 353.2244	334.2079[C_20_H_30_O_4_], 316.198[C_20_H_28_O_3_], 181.0866[C_10_H1_3_O_3_]	352.2176, 352.2166	333.2071[C_20_H_29_O_4_], 307.1914[C_18_H_27_O_4_]	− 3.8969/2.8182	245		*	^59^
5	6.74	Boscartin J C/C(CC/C = C1/C) = C\C[C@]2(C(C)C)CC[C@](O2)(C)[C@H](O)C[C@@H]1O	C_20_H34O_3_	323.2563, 323.2581	305.2480[C_20_H_33_O_2_], 287.2345[C_20_H_31_O]	321.2107, 321.2060	303.2893[C_18_H_39_O_3_], 199.1694[C_12_H_29_O_2_]	− 5.4270	238 277		*	^60^
7	7.78	Incensole oxide CC(C)[C@@]12CC[C@](C)([C@@H](O)CC/C(C) = C/CC[C@](O3)(C)C3([H])C2)O1	C_20_H_34_O_3_	323.2582, 323.2561	305.2461[C_20_H_33_O_2_], 287.2353[C_20_H_31_O], 263.1628[C_16_H_23_O_3_], 151.1118[C_10_H_15_O]			0.3318	245	*	*	^59^
8	8.29	Boscartin C CC(C)[C@@]12CC[C@](C)(C(O)CC/C(C) = C/C(O)C[C@]3(C)C(O3)([H])C2)O1	C_20_H_34_O_4_	339.2531, 339.2530	303.2318[C_20_H_31_O_2_], 285.2211[C_20_H_29_O], 151.1119[C_10_H_15_O]	337.2383, 337.2373	293.2118[C_18_H_29_O_3_]	0.1937/2.8029	245		*	^59^
10	8.34	Boscartins P [H][C@@]12C[C@@]3(CC[C@@](C)(O3)[C@]3([H])CC[C@@](C)(O)[C@]([H])(CC[C@@]1(C)O2)O3)C(C)C	C_20_H_34_O_4_	339.2526, 339.2529	321.2422[C_20_H_33_O_3_], 303.2321[C_20_H_31_O_2_], 285.2202[C_20_H_29_O], 251.1997[C_16_H_27_O_2_], 151.1119[C_10_H_15_O]	337.2383, 337.2373	319.1923[C_19_H_27_O_4_],	− 1.2456/2.8029	245, 277		*	^1^
11	8.79	Boscartins Q [H][C@@]12C[C@@]3(CC[C@@](C)(O3)[C@H](CC\C(C) = C\[C@@H](O)C[C@@]1(C)O2)OC(C) = O)C(C)C	C_22_H_36_O_5_	381.2488, 381.2499	303.2324[C_20_H_31_O_2_], 137.0964[C_9_H_13_O]	379.2495, 379.2479	347.1853[C_20_H_27_O_5_], 245.1551[C_16_H_21_O_2_]	4.1676	245		*	^1^
12	8.90	Boscartins X CC(C)[C@@]12CC[C@@](C)(O1)[C@H](CC\C(C) = C\CCC(= C)[C@H](O)C2)OC(C) = O	C_22_H_36_O_4_			363.2373, 363.2373	317.2119[C_20_H_29_O_3_], 273.1853[C_18_H_25_O_2_]	− 0.1711	245		*	^1^
13	9.02	Boscartins AA CC(C)[C@@]12CC[C@@](C)(O1)[C@H](CC[C@](C)(O)\C = C\C(= O)\C(C) = C\C2)OC(C) = O	C_22_H_34_O_5_	379.2458, 379.2479	361.2358[C_22_H_33_O_4_], 319.2269[C_20_H_31_O_3_], 301.2162[C_20_H_29_O_2_], 215.1432[C_15_H_19_O]	377.2334, 377.2323	315.2322[C_21_H_31_O_2_],	− 5.6495/2.9619	245		*	^1^
16	9.20	Boscartins Z CC(C)[C@@]12CC[C@@](C)(O1)[C@H](CC[C@@](C)(O)\C = C\C(= O)\C(C) = C\C2)OC(C) = O	C_22_H_34_O_5_	379.2480, 379.2479	319.2285[C_20_H_31_O_3_], 301.2159[C_20_H_29_O_2_], 243.1738[C_17_H_23_O], 215.1429[C_15_H_19_O]	377.2331, 377.233	333.2444[C_21_H_33_O_3_], 265.1505[C_15_H_21_O_4_]	0.1442/2.2338	245		*	^1^
19	9.35	Boscartin D CC(C)[C@@]12CC[C@](C)(C(O)CC/C(C = O) = C/CC[C@]3(C)C(O3)([H])C2)O1	C_20_H_32_O_4_	337.2377, 337.2373	319.2261[C_20_H_31_O_3_], 301.2160[C_20_H_29_O_2_], 283.2061[C_20_H_27_O]			0.9931	245		*	^59^
21	9.65	(rel)-(1S,5R,7E,11E)-1-isopropyl-8,12-dimethyl-4- methylenecyclotetradeca-7,11-diene-1,5-diol CC(C)[C@]1(O)CC\C(C) = C\CC\C(C) = C\C[C@@H](O)C(= C)CC1	C_20_H_34_O_2_	306.2546, 306.2553	288.2398[C_20_H_32_O]			− 2.2633	245, 274	*	*	^61^
22	9.85	Boscartin B CC(C)[C@@]12CC[C@](C)([C@]3([H])CC[C@](O3)(C)C(O)CC4(O[C@@]4(C)C(O)C2)[H])O1	C_20_H_34_O_5_	355.2473, 355.2479	337.2370[C_20_H_33_O_4_], 319.2267[C_20_H_31_O_3_], 301.2146[C_20_H_29_O_2_]	353.2334, 353.2323	335.2221[C_20_H_31_O_4_], 309.2431[C_19_H_33_O_3_], 141.0907[C8H13O2]	3.1631/ − 1.7360	245	*	*	^59^
23	9.98	boscartin A O[C@H]1[C@](O2)(C)CC[C@]2([H])/C(C) = C\C[C@@H](O)[C@](O3)(C)CC[C@]3(C(C)C)C1	C_20_H_34_O_4_	339.2519, 339.2530	321.2425[C_20_H_33_O_3_], 303.2314[C_20_H_31_O_2_], 285.2217[C_20_H_29_O], 151.1119[C_10_H_15_O]	337.2283, 337.2373	251.1654[C_12_H_23_O_3_], 139.0751[C_8_H_11_O_2_]	2.8029	245, 306/249	*	*	^59^
30	10.92	(1S,3E,7E,11S,12R)-1-isopropyl-4,8,12- trimethyl-11-hydroxyl-15-oxabicyclo[10.2.1] pentadeca-3,7-dien-9-one CC(C)[C@@]12CC[C@@](C)(O1)[C@@H](O)CC(= O)\C(C) = C\CC\C(C) = C\C2	C_30_H_32_O_2_	321.2419, 321.2424	289.2521[C_20_H_33_O]			− 1.7731	245	*		^62^
42	13.38	Incensole CC(C)[C@@]12CC[C@](C)([C@@H](O)CC/C(C) = C/CC/C(C) = C/C2)O1	C_30_H_34_O_2_	307.2643, 307.2632	289.2521[C_20_H_33_O]			3.8612	252	*	*	^63^
43	13.49	Incensol Acetate CC([C@]12CC[C@](O2)(C)[C@H](CC/C(C) = C/CC/C(C) = C/C1)OC(C) = O)C	C_22_H_36_O_3_	349.2732, 349.2737	289.2520[C_20_H_33_O]			− 1.5995	252	*	*	^63^
Group B: prenylaromadendrane-type diterpenoids												
6	6.79	Boscartol M [H][C@@]12C(CCC(= C)[C@]3([H])CC[C@](C)(O)[C@@]13[H])[C@]2(C)C(O)\C = C\C(C) = O	C_19_H_28_O_3_	305.2107, 305.2111	287.2002[C_19_H_27_O_2_], 269.1889[C_19_H_25_O]	303.1996, 303.1955	285.0402[C_19_H_25_O_2_]	− 1.3020	245	*		^60^
9	8.25	Boscartol K [H][C@@]12CCC(= C)[C@]3([H])CC[C@](C)(O)C3[C@]1([H])C2(C)[C@H](O)C1OC(= O)C(C) = C1	C_20_H_28_O_4_	333.2046, 333.2060	315.1955[C_20_H_27_O_3_], 297.1836[C_20_H_25_O_2_]	331.1914, 331.1904	313.1810[C_20_H_25_O_3_]	− 4.3398/3.0811	245		*	^60^
14	9.13	Boscartol P [H][C@@]12CCC(= C)[C@]3([H])CC[C@](C)(O)[C@@]3([H])[C@]1([H])[C@@]2(C)CC\C = C(\C)COC(C) = O	C_22_H_34_O_3_	347.2581, 347.2581	311.2367[C_22_H_31_O], 243.2315[C_15_H_31_O_2_]			0.000	245	*		^64^
17	9.29	Boscarterol A [H][C@@]12CCC(= C)[C@]3([H])CC[C@](C)(O)C3C1[C@@]2(C)\C = C\C = C(\C)CO	C_20_H_30_O_2_	303.2318, 303.2319	285.2214[C_20_H_29_O], 245.1908[C_17_H_25_O]	299.2017, 299.2006 [M-2H]	285.2899[C_20_H_29_O],	− 0.0484/3.8923	245	*		^65^
20	9.46	Boscarterol F [H][C@@]12CCC(= C)[C@]3([H])CC[C@](C)(O)[C@@]3([H])[C@]1([H])[C@@]2(C)\C = C\C = C(\C)C = O	C_20_H_28_O_2_	300.2085, 300.2084	282.1932[C_20_H_26_O]			− 0.3331	249	*		^60^
29	10.64	Boscarterol G [H][C@@]12CCC(= C)[C@]3([H])CC[C@](C)(O)C3[C@]1([H])[C@@]2(C)[C@@]1([H])[C@H](O)C = C(C)C1 = O	C_20_H_28_O_3_			315.1962, 315.1955	297.2377[C_18_H_33_O_3_], 271.2072[C_19_H_27_O]	2.3034		*	*	^65^
37	12.87	Olibanumol D	C_20_H_30_O	286.2293, 286.2292	268.2136, 258.2307			− 0.6987	251	*	*	^66^
45	13.74	Boscartol A [H][C@@]12CCC(= C)[C@]3([H])CC[C@](C)(O)[C@@]3([H])[C@]1([H])[C@@]2(C)C = CC = C(C)CO	C_20_H_30_O_2_	303.2312, 303.2319	285.2215[C_20_H_29_O]			− 2.2626	252	*		^65^
46	13.76	Boscartol C [H][C@@]12CCC(= C)[C@]3([H])CC[C@](C)(O)[C@@]3([H])[C@]1([H])[C@@]2(C)CC = CC(C)(C)O	C_20_H_32_O_2_	305.2476, 305.2475	287.2359[C_20_H_31_O]			0.1697	252	*		^65^
47	13.80	Boscartol G [H][C@@]12CCC(= C)[C@]3([H])CC[C@](C)(O)C3[C@]1([H])[C@@]2(C)[C@@]1([H])[C@H](O)C = C(C)C1 = O	C_20_H_28_O_3_	317.2112, 317.2111	299.2011[C_20_H_27_O_2_], 281.1895[C_20_H_25_O]	315.1965, 315.1955	297.2439[C_18_H_33_O_3_], 271.2071[C_19_H_27_O]	0.3827/3.3693	252	*		^65^
Group C: polyphenolics and derivatives												
1	2.16	Quinic acid	C_7_H_12_O_6_			191.0552, 191.0550	173.0440[C_7_H_9_O_5_], 85.0279[C_4_H_5_O_2_]	0.9239	220	*	*	Ms-Dial
2	2.72	Gallic acid	C_7_H_6_O_5_	171.0289, 171.0288	153.0184[C_7_H_5_O_4_], 127.0393[C_6_H_7_O_3_]	169.0131, 169.0131	125.0229[C_6_H_5_O_3_], 59.0122[C_2_H_3_O_2_]	− 0.1609/0.3190	202,241, 267			Ms-Dial
15	9.14	Fisetin	C_15_H_10_O_6_			285.0403, 285.0394	171.1019[C_9_H_15_O_3_], 151.0024[C_7_H_3_O_4_], 127.1116[C_8_H_15_O]	3.4373	245	*		Ms-Dial
Group D: triterpenes belong to tirucallane-type												
24	9.97	Sacraoic acid C [H][C@@](CCC = C(C)C)(C(O) = O)[C@]1([H])CC[C@]2(C)C3 = C([C@@H](O)C[C@@]12C)[C@@]1(C)CC[C@@H](O)C(C)(C)[C@]1([H])CC3 = O	C_30_H_46_O_5_	487.3425, 487.3418	451.3221[C_30_H_43_O_3_], 316.3476[C_30_H_44_O_2_]	485.3280, 485.3262	441.3381[C_29_H_45_O_3_], 383.2964[C_26_H_39_O_2_]	3.8159	245, 306	*	*	^67^
25	10.17	Sacraoic acid D [H][C@@](CCC = C(C)C)(C(O) = O)[C@]1([H])CC[C@]2(C)C3 = C(C(= O)C[C@@]12C)[C@@]1(C)CC[C@H](O)C(C)(C)[C@]1([H])CC3 = O	C_30_H_44_ O_5_	485.3256, 485.3262	467.3158[C_30_H_43_O_4_], 449.3051[C_30_H_41_O_3_]	483.3117, 483.3105	439.3220[C_29_H_43_O_3_], 381.2796[C_26_H_37_O_2_]	3.1260/ − 1.0887	245, 310	*	*	^67^
26	10.04	Spirosacraoic acid B [H][C@@](CCC = C(C)C)(C(O) = O)[C@]1([H])CC[C@]2(C)C(= O)[C@@]3(CC[C@@]12C)[C@H](O)C[C@]1([H])[C@]3(C)CCC(= O)C1(C)C	C_30_H_46_O_5_	487.4325, 487.3418	469.3294[C_30_H_45_O_4_], 423.3261[C_29_H_43_O_2_]	485.3275, 485.3262	467.3161[C_30_H_43_O_4_], 441.3384[C_29_H_45_O_3_], 340.2454[C_24_H_36_O]	1.4944/2.7470	245, 306	*	*	^67^
27	10.12	Boscartene L [H][C@@]1([C@@]2(CC[C@H](C(C)(O)C)OC2 = O)[H])CC[C@@]3(C4 = C([C@]5(CCC(C(C)([C@@]5(CC4 = O)[H])C) = O)C)CC[C@@]13C)C	C_30_H_44_O_5_	485.3261, 485.3262	467.3177[C_30_H_43_O_4_], 451.3221[C_30_H_43_O_3_], 421.3103[C_29_H_41_O_2_]	483.3112, 483.3105	465.3025[C_30_H_41_O_4_], 365.2841[C_26_H_37_O], 369.2425[C_24_H_33_O_3_], 255.2324[C_16_H_31_O_2_]	− 0.1455/1.4211	245, 277	*	*	^68^
28	10.35	Boscartene M [H][C@@]1([C@@]2(CC[C@H](C(C)(O)C)OC2 = O)[H])CC[C@@]3(C4 = C([C@]5(CCC(C(C)([C@@]5(CC4 = O)[H])C) = O)C)C(C[C@@]13C) = O)C	C_30_H_42_O_6_	499.3043, 499.3054	481.2960[C_30_H_41_O_5_], 435.2896[C_29_H_39_O_3_], 369.2424[C_24_H_33_O_3_]	497.2912, 497.2898	453.3017[C_29_H_41_O_4_], 401.2329[C_24_H_33_O_5_], 301.1808[C_19_H_25_O_3_]	− 2.3137/2.8208	245, 306	*	*	^68^
31	11.84	Boscartene N [H][C@]1(CC[C@@]2(C3 = C([C@]4(CCC(C(C)([C@@]4(CC3 = O)[H])C) = O)C)CC[C@@]12C)C)[C@@]5([H])CC(O[C@H]5/C = C(C)/C) = O	C_30_H_42_O_4_	467.3163, 467.3156	449.3051[C_30_H_41_O_3_], 403.2992[C_29_H_39_O],	465.3006, 465.2999	450.2787[C_29_H_38_O_4_], 406.2883[C_28_H_38_O_2_], 369.2423[C_24_H_33_O_3_]	1.6240/1.4881	249	*	*	^68^
34	12.09	Boscartene E [H][C@]1(C[C@@H](OC1 = O)C = C(C)C)[C@]1([H])CC[C@]2(C)C3 = C([C@@H](O)C[C@@]12C)[C@@]1(C)CCC(= O)C(C)(C)[C@]1([H])CC3 = O	C_30_H_42_O_5_	483.3108, 483.3105	437.3052[C_29_H_41_O_3_], 419.2934[C_29_H_39_O]	481.2956, 481.2949	437.3065[C_29_H_41_O_3_], 385.2383[C_24_H_33_O_4_], 368.2357[C_24_H_32_O_3_]	− 0.9783/1.5426	249		*	^69^
35	12.31	Boscartene C [H][C@]1(CC[C@]2(C)C3 = CC[C@@]4([H])C(C)(C)C(= O)CC[C@]4(C)C3 = CC[C@@]12C)[C@]1([H])CC[C@@H](OC1 = O)C(C)(C)O	C_30_H_44_O_4_	469.3312, 469.3312	451.3221[C_30_H_43_O_3_], 422.3123[C_29_H_42_O_2_]	466.3163, 466.3156 [M-2H]	423.3271[C_29_H_43_O_2_], 371.2592[C_24_H_35_O_3_]	0.0031/1.6240	429	*	*	^69^
36	12.55	Boscartene B [H][C@]1(CC[C@]2(C)C3 = C(CC[C@@]12C)[C@@]1(C)CCC(= O)C(C)(C)[C@]1([H])CC3)[C@]1([H])CC[C@@H](OC1 = O)C(C)(C)O	C_30_H_46_O_4_	471.3471, 471.3469	453.3347[C_30_H_45_O_3_], 407.3285[C_29_H_43_O]	468.3323, 468.3312 [M-2H]	451.3219[C_30_H_43_O_3_], 407.3318[C_29_H_43_O], 371.2592[C_24_H_35_O_3_]	0.4679/2.2139	249	*	*	^69^
Group E: pentacyclic triterpenes belong to ursane type												
18	9.32	Madecassic acid O = C([C@]12CC[C@@H](C)[C@H](C)[C@@]1([H])C3 = CCC4[C@@]5(C)C[C@@H](O)[C@H](O)[C@@](C)(CO)[C@]5([H])[C@H](O)C[C@@]4(C)[C@]3(C)CC2)O	C_30_H_48_O_6_			503.3385, 503.3367	485.3274[C_30_H_45_O_5_], 459.3500[C_29_H_47_O_4_], 401.3072[C_26_H_41_O_3_]	3.5470	245			Reaxys
39	13.20	Olibanumol G O[C@@H]1C(C)(C)[C@]2(O)CC[C@@]3(C)[C@]4(C)CC[C@](CC[C@H]5C(C) = C)(C)[C@]5([H])C4CC[C@]3([H])[C@@]2(C)CC1	C_30_H_50_O_2_	442.3782, 442.3805	424.3640[C_30_H_48_O]			− 5.2624	252	*		^66^
41	13.36	*β* -Boswellic acid [H][C@@]12[C@@H](C)[C@H](C)CC[C@]1(C)CC[C@]1(C)C2 = CC[C@]2([H])[C@@]3(C)CC[C@@H](O)[C@](C)(C(O) = O)[C@]3([H])CC[C@@]12C	C_30_H_48_O_3_	457.3681, 457.3676	439.3214[C_29_H_43_O_3_], 411.3228[C_28_H_43_O_2_]	455.3527, 455.3520	409.3087[C_28_H_41_O_2_]	1.1187/1.5808	256	*		^70^
44	13.72	11-Keto-*β* -boswellic acid [H][C@@]12[C@@H](C)[C@H](C)CC[C@]1(C)CC[C@]1(C)C2 = CC(= O)[C@]2([H])[C@@]3(C)CC[C@@H](O)[C@](C)(C(O) = O)[C@]3([H])CC[C@@]12C	C_30_H_46_O_4_	471.3470, 471.3469	407.3328[C_29_H_43_O]	469.3320, 469.3312	407.3329[C_29_H_43_O]	0.3384/1.5636	252	*	*	^70^
49	14.91	Ursolic acid [H][C@@]12[C@@H](C)[C@H](C)CC[C@@]1(CC[C@]1(C)C2 = CC[C@]2([H])[C@@]3(C)CC[C@H](O)C(C)(C)[C@]3([H])CC[C@@]12C)C(O) = O	C_30_H_48_O_3_			455.3532, 455.3520	311.1710	2.7202	256	*		^71^
31	11.11	Asiatic acid O = C([C@]12CC[C@@H](C)[C@H](C)[C@@]1([H])C3 = CCC4[C@@]5(C)C[C@@H](O)[C@H](O)[C@@](C)(CO)[C@]5([H])CC[C@@]4(C)[C@]3(C)CC2)O	C_30_H_48_O_5_			487.3430, 487.3418	469.3322[C_30_H_45_O_4_], 373.2738[C_24_H_37_O_3_]	2.4337	249	*	*	Ms-dial
Group F: triterpene belong to oleanane Type												
32	11.44	Oleanolic acid [H][C@@]12CC(C)(C)CC[C@@]1(CC[C@]1(C)C2 = CC[C@]2([H])[C@@]3(C)CC[C@H](O)C(C)(C)[C@]3([H])CC[C@@]12C)C(O) = O	C_30_H_48_O_3_	457.3687, 457.3678	411.3257[C28H43O2]			1.7193	249	*		Ms-dial KNApSAcK
33	11.95	Beta-Elemonic acid C/C(C) = C\CCC(C(O) = O)C1CCC2(C)C1(C)CCC3 = C2CCC4C3(C)CCC(C4(C)C) = O	C_30_H_46_O_3_	455.3542, 455.3520	437.3424[C_30_H_45_O_2_], 419.33324[C_30_H_43_O]	453.3004, 453.2999	435.2917[_C29_H_39_O_3_], 409.3105[C_28_H_41_O_2_]	4.7978/0.9890	249		*	^70^
40	13.23	Elemonic acid [H][C@@](CCC = C(C)C)(C(O) = O)[C@]1([H])CC[C@]2(C)C3 = C(CC[C@@]12C)[C@@]1(C)CCC(= O)C(C)(C)[C@]1([H])CC3	C_30_H_46_O_3_	454.3445, 454.3441	436.3305[C_30_H_44_O_2_], 408.3334[C_29_H_44_O]	452.3375, 452.3363	435.3270[C_30_H_43_O_2_]	0.8731/2.6529	252			^70^
34	12.07	Maslinic acid [H][C@@]12CC(C)(C)CC[C@@]1(CC[C@]1(C)C2 = CC[C@]2([H])[C@@]3(C)C[C@@H](O)[C@H](O)C(C)(C)[C@]3([H])CC[C@@]12C)C(O) = O	C_30_H_48_O_4_			471.3477, 471.3469	427.3571[C_29_H_47_O_2_], 385.3084[C_26_H_41_O_2_]	1.7628		*		Ms-dial
Group G: triterpene belong to lupane type												
37	12.69	3*β* ,20-dihydroxylupane-28-oic acid [H][C@]12[C@@H](CC[C@@]1(CC[C@]1(C)[C@]2([H])CC[C@]2([H])[C@@]3(C)CC[C@H](O)C(C)(C)[C@]3([H])CC[C@@]12C)C(O) = O)C(C)(C)O	C_30_H_50_O_4_	475.3786, 475.3782	457.3683[C_30_H_49_O_3_], 411.3262[C_28_H_43_O_2_], 335.2579[C_21_H_35_O_3_]	473.3604, 473.3625	–	− 1.2927/ − 4.4223	249	*		^72^
38	12.91	Alphitolic acid [H][C@]12[C@@H](CC[C@@]1(CC[C@]1(C)[C@]2([H])CC[C@]2([H])[C@@]3(C)C[C@@H](O)[C@H](O)C(C)(C)[C@]3([H])CC[C@@]12C)C(O) = O)C(C) = C	C_30_H_48_O_4_			471.3480, 471.3469	407.3301[C_29_H_43_O]	2.4102	252	*	*	Ms-dial
Group H: fatty acids												
48	13.81	Oleic acid, methyl ester	C_19_H_36_O_2_			295.2640, 295.2632	245.2508[C_15_H_33_O_8_]	2.8812	252	*	*	Ms-dial

The preliminary identification was made using the entire extract of BC and the ethyl acetate fraction. The letters signify the compounds that were compared with existing literature, the compounds that were identified using the KNApSAcK database, the compounds that were identified using MS_Dial, and the compounds that were identified using Metlin.

Table 2 and Fig. 1 have illustrated the anti-inflammatory effects of potential identified compounds found in BC-Resins. Cembrane-type diterpenoids have been shown to significantly inhibit the production of TNF-α and IL-6, as well as the expression of iNOS, COX-2, and p-NF-κB. Additionally, this compound has displayed a strong inhibitory potential against the inflammatory agent 5-lipoxygenase^73,74^. Prenylaromadendrane-type diterpenoids have demonstrated powerful inhibitory effects on the production of NO^60^. Triterpenes, including tirucallane, ursane, oleanane, and lupane types, have the ability to prevent the production of leukotriene in neutrophilic granulocytes by inhibiting 5-lipoxygenase. Moreover, certain boswellic acids can hinder the growth of cancer cell lines, induce apoptosis, block topoisomerases, and inhibit elastase in leukocytes^72^. Polyphenolics and their derivatives display inhibitory effects on phospholipase A2 (PLA2), cyclooxygenase (COX), and lipoxygenase (LOX), leading to a reduction in the synthesis of prostaglandins (PGs) and leukotrienes (LTs), thereby exhibit anti-inflammatory activity^75^.

### Preparation of nano sponges

Nano sponges based on cyclodextrin have become more well-known recently because of their special qualities. These sponges especially improve the stability and apparent solubility of medications by creating inclusion complexes through the inner nanocavities of cyclodextrins and non-inclusion complexes through the gaps in the cross-linked polymeric network of the sponges. Cyclodextrin-based nanostructures (NSs) such as HP*β* -CD can provide a promising class of cross-linked polymers with an amazing three-dimensional architecture consisting of hydrophilic and hydrophobic nanosized pores that can effectively encapsulate a wide range of drugs and improve the solubility of less soluble drugs^76^. In the present study, NSs were prepared by ultrasound assisted technique. This method produces NSs with spherical shape and uniform in size^77^. Table 3 shows the formulation components as well as the findings reached from the evaluation of the responses. DEX NSs was created in the current study utilizing HP*β* -CD as the polymer and DPC as the crosslinker in varied ratios (1:5, 3:1, 4:1, and 5:1). NSs were effectively prepared in all of the formulations examined.

**Table 3:** Composition, entrapment efficiency and physico-chemical properties of DEX NSs and the optimized NSs formulation.

Code	Drug	Molar ratio			EE% ± SD	VS(nm) ± SD	PDI	ZP (mV) ± SD
		2-Hydroxy propyl-β-cyclodextrin (2HP-β-CD)	Polymerized-β-cyclodextrin (Epi-β-CD)	Di-phenyl carbonate (DPC)				
D1	DEX	1	–	5	99.95 ± 1.60	166.8 ± 26.3	0.524	− 27 ± 6.26
D2	DEX	3	–	1	98.52 ± 0.07	109.1 ± 32.4	0.612	− 22.32 ± 1.15
D3	DEX	4	–	1	99.64 ± 1.40	105.9 ± 15.9	0.371	− 20.53 ± 2.13
D4	DEX	5	–	1	99.23 ± 0.20	160.8 ± 24.3	0.604	− 26.53 ± 3.79
D5	DEX	1	5	–	100% ± 6.24	74.04 ± 55.4	0.420	− 29.92 ± 5.68
P1	BC extract	1		5	87.39% ± 1.99	244 ± 17.54	0.507	− 25.4 ± 4.13
P2	BC extract	1	5	–	98.56% ± 2.88	359 ± 30.23	0.458	− 28 ± 3.87

### Encapsulation efficiency

The EE% values for each DEX NSs formulation (D1–D4) are displayed in Table 3. All formulations had EE% values between 98.52 ± 0.07 and 99.64 ± 1.40%. The success of DEX NS preparation was confirmed by the obvious high encapsulation efficiency (EE %) of all prepared NSs. The excellent crosslinking between HP*β* -CD and DPC, which permits a large inclusion of DEX salt in the NSs matrix and cyclodextrin cavity, may be responsible for the high EE% of the generated NSs^78,79^. Along with the inclusion of DEX salt in the porous matrix of the Nano sponge, it is also responsible for entirely trapping the drug molecules as an inclusion complex inside the hydrophobic host cyclodextrins' cavities, which are surrounded by hydrophilic nanochannels^80,81^.

### Vesicle size, polydispersity index and zeta potential

The analysis of DEX NSs revealed that all produced NSs were in the nanosized range, with formulation sizes ranging from 105.9 ± 15.9 to 166.8 ± 26.3 nm (Table 3). It has been observed that formulations comprising a higher molar ratio of cross linker exhibited PS greater than those comprising a lower molar ratio of cross linker. This finding is in good agreement with previous reports^82,83^, where the increase in crosslinker molar ratio resulted in a larger PS. Additionally, the data supported low polydispersity index (PDI) values of 0.371 to 0.612, which indicated a uniform and constrained vesicle size distribution^84^. Table 3 also shows that all of the formulations under investigation had a negatively charged zeta potential; this could be because of the free hydroxyl groups of *β* CD and 2-HP*β* CD, as well as the carbonyl groups of DPC^85,86^. All formulations under investigation had absolute ZP values more than 20, which are more than enough to maintain the individual particles' separation from one another by electrostatic repulsion. Thus, scattered particles are therefore physically stable.

### Selection of the optimized DEX salt and ethyl acetate plant extract NSs formulations

According to the results of EE%, PS, PDI and ZP, the best molar ratio between HP*β* -CD and DPC was (1:5) (D1). The same molar ratio HP *β*-CD: DPC (1:5), was used for the preparation of Plant extract NSs (P1) and the same molar ratio was used for the preparation of NSs using HP *β*-CD and EPI-*β*-CD in the ratio of (1:5) and loading of DEX and ethyl acetate extraction in the new formulations (D5 and P2). The λ_max_ of the plant extract was found to be at 252nm. The EE%, PS, PDI and ZP of the new formulations were estimated and demonstrated in Table 3. Results revealed that new formulations D5, P1 and P2 exhibited high EE% (87.39% ± 1.99 to 100 ± 6.24), with particle size in the nano size range, low PDI and suitable ZP value. It was noticed that there was non-significant difference (p ≥ 0.001) in EE% between D1 and D5 as use of EPI-*β*-CD instead of DPC doesn’t affect EE% of DEX salt, On the other hand the use of EPI-*β*-CD in the preparation of ethyl acetate plant extract NSs lead to a significant increase (p ≤ 0.001) in EE%. Since few years, studies have been carried out toward the use of epichlorohydrin- *β*-cyclodextrin (EPI- *β*-CD) polymer for the preparation of nano sponges^87,88^, as this polymerized form of *β*-cyclodextrin remains within the cavity structure of *β*-CD providing capability of forming inclusion complexes with a variety of guest molecules which can lead to increase the loading capacity of NSs^89^.

### Characterization of the optimized formulations

#### Surface morphology

##### Transmission *electron* microscopy (TEM)

**Figure 2:**
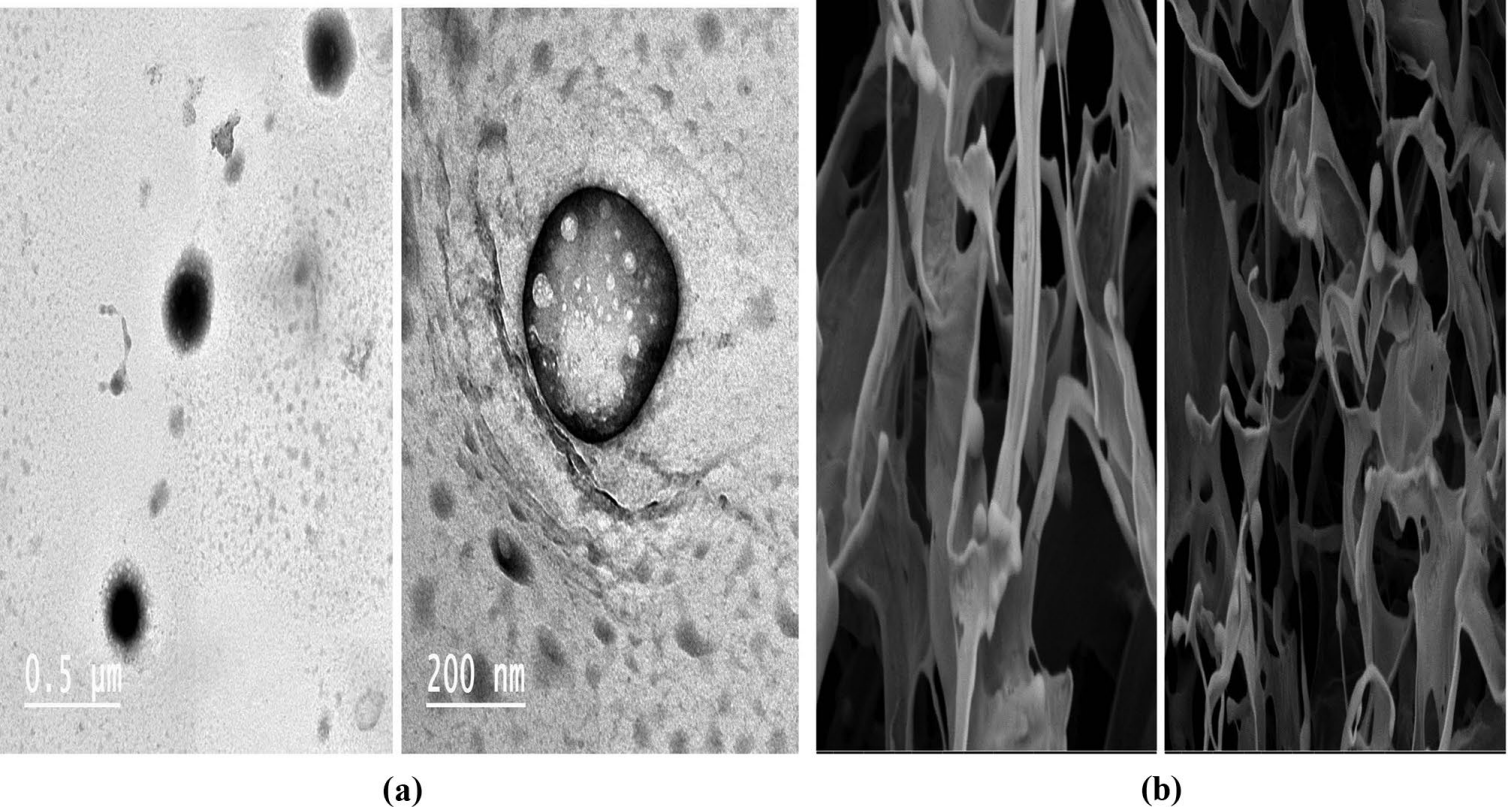
(**a**) TEM micrographs of Nano sponges optimized formulations, (**b**) SEM micrographs of Nano sponges optimized formulations.

Figure 2a depicts the morphology of NSs. The NSs have been found to be round and uniform, with no drug crystals on the surface. According to the figures, the NSs created using the ultrasonic assisted process have a uniform size distribution, crystallinity, and a porous character^90^.

##### Scanning electron microscopy (SEM)

The SEM images of NSs were illustrated in Fig. 2b. SEM analysis of the prepared NSs revealed nano-sized spherical particles having multiple pores on their surface^91^. There was no residual crystals from the drugs indicating the complete encapsulation of drugs in the polymer^92^.

#### Fourier transform infrared spectroscopy analysis

**Figure 3:**
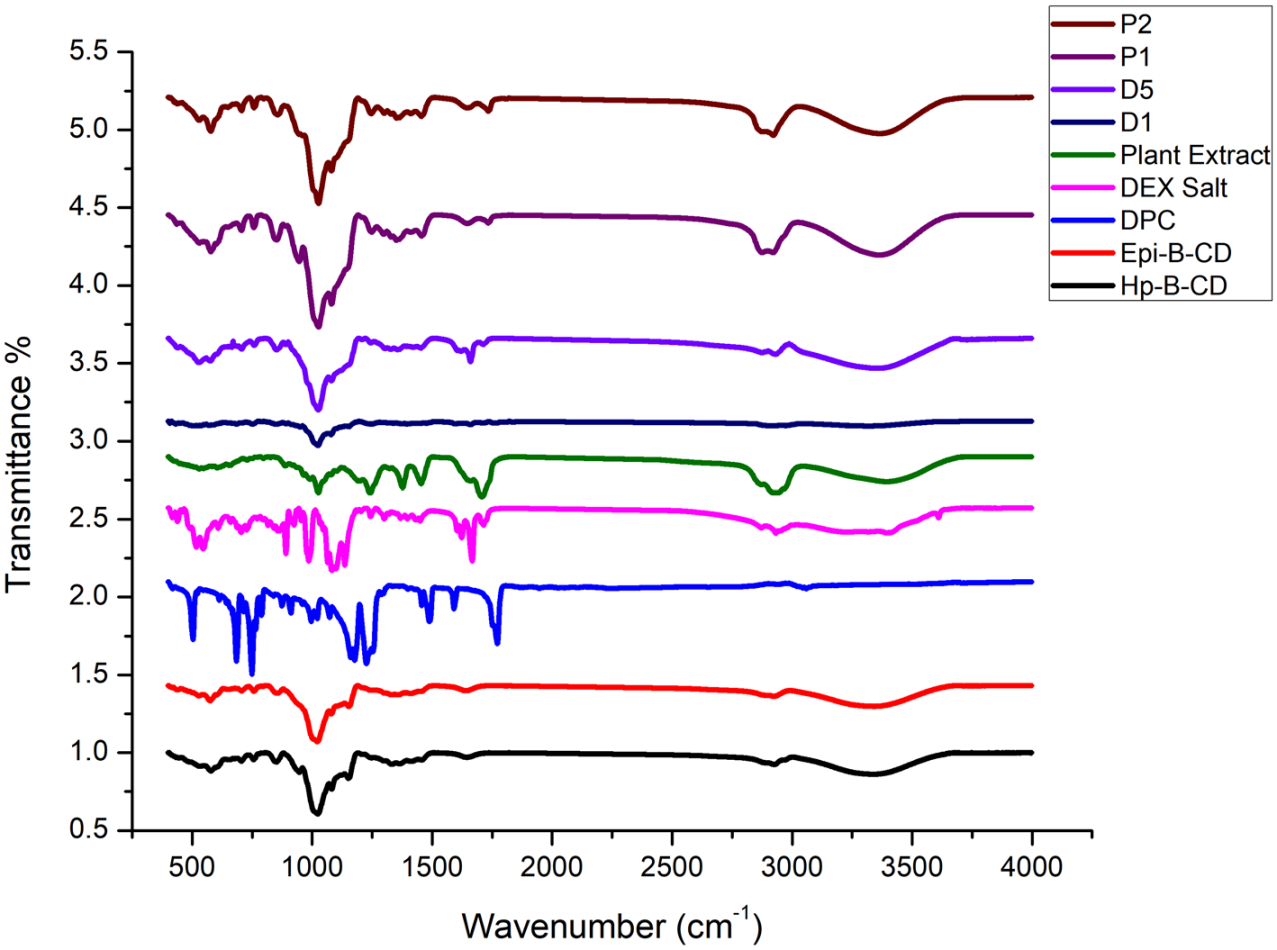
IR spectrum of different Nano sponges components and optimized formulations.

The interactions between pharmaceuticals and excipients were investigated by comparing the FTIR spectra of pure components with drug-loaded Nano sponges (D1, D5, P1 and P2) (Fig. 3). The FTIR spectra of HP-*β*-CD showed prominent absorption bands at 3415 cm^−1^ (O–H stretching), 2929 cm^−1^ (C–H stretching), 1645 cm^−1^ (H–O-H bending), 1157 cm^−1^ (C–O stretching), and 1031 cm^−1^ (C–O–C stretching)^93,94^. FTIR spectrum bands for DPC revealed distinctive absorbance band at 1753 cm^-1^ for carbonate bond^95^. Epichlorohydrin-*β*-cyclodextrin (EPI- *β* -CD) has distinctive peaks for epichlorohydrin at 1288.36, 1249.79, and 721.33 cm^−1^, respectively, as well as a CH_2_Cl wagging band and C–Cl stretching in its IR spectra. The OH, CH_2_, and C–O–C stretching vibrations, which are approximately 3452.34, 3411.84, 3271.05, 2933.53, and 1099.35 cm^−1^, respectively, confirm that cyclodextrin is present in the structure^96^.

The FTIR spectra of DPC showed distinctive peaks at 1513, 1509, 1480, 1397, 1314, 1312, 1214, 1210, 1176 and 1120 cm^–1^ that can be assigned to C=H bending vibrations. The molecule containing carbonyl group shows strong absorption band for C=O stretching vibrations at the region 1850 and 1550 cm^–1^^97^.

DEX exhibited distinct absorbance bands at 1706, 1660, and 1616 cm^–1^, which were attributed to –C=O stretching vibrations connected to C3-cyclic and C20 carbonyl groups, as well as double bond context coupled to –C=O bonds. Furthermore, two more different absorption bands of 3468 cm^-1^ and 1270 cm^–1^ were realized due to the stretching ambiences of the O–H and C–F bonds, respectively^98,99^.

The comparative nature of the IR spectrum of BC ethyl acetate plant extract can be observed when compared to Boswellic acids. It is worth noting that the spectrum exhibits characteristic peaks at specific wavenumbers, namely 3437 cm^–1^ (indicative of OH stretching), 2932 cm^–1^ (associated with C–H stretching), 1697 cm^–1^ (highlighting C=O stretching of aryl acid), 1453 cm^-1^ (pertaining to C–H bending), 1375 cm^–1^ (linked to COO symmetric stretching of carboxylates), 1240 cm^–1^ (relating to C–CO–C stretching of aryl ketone), as well as 1025 cm^–1^ and 988 cm^–1^ (both associated with ring structures of cyclohexane)^100^.

In the distinctive peaks of DEX or ethyl acetate plant extract, the IR spectrum of the optimized Nano sponge formulations (D1, D5, P1 and P2) revealed a shifting and diminished intensity. This shift in the characteristic peaks may be explained by the presence of physical interactions between drugs and various NS elements, such as Van der Wall bonds, hydrogen bonds, or dipole interactions, without any chemical changes to the drugs' structure after encapsulation, which can result in the best possible entrapment of DEX or ethyl acetate plant extract in Nano sponges^85,101,102^.

### In vitro release study

CD-based NSs can be a useful technique for delivering medications in a sustained manner. Drug encapsulation in a crosslinked NSs structure allows for prolonged drug administration, allowing for lower doses, less side effects, and changed pharmacokinetics. These characteristics can be used to improve medicine distribution^103^

**Figure 4:**
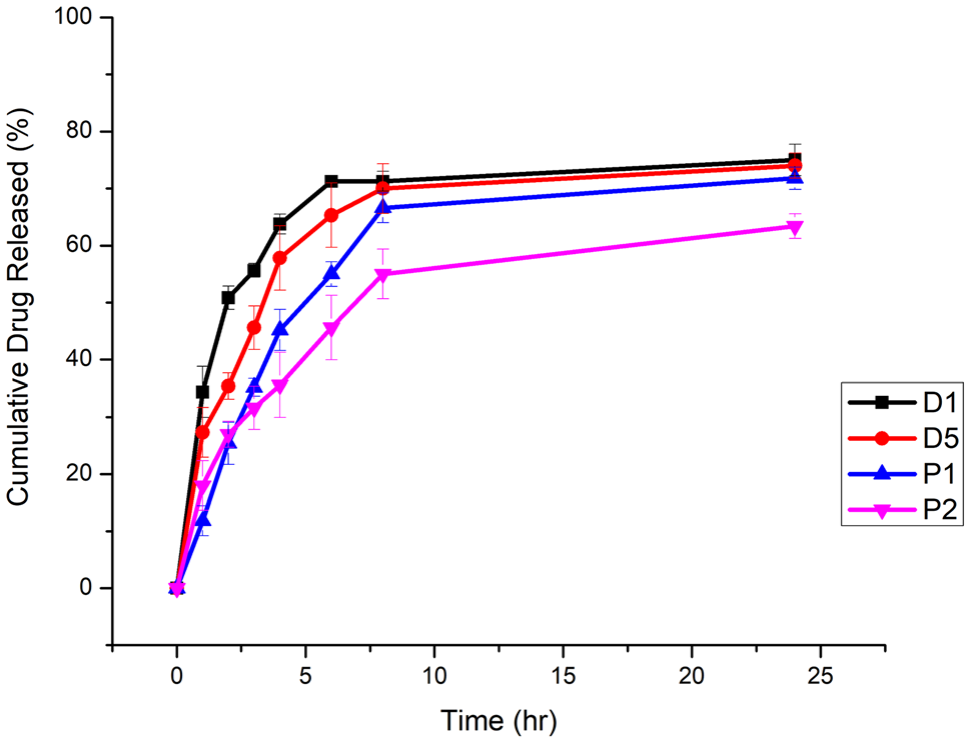
In-vitro release profiles of drugs from Nano sponges optimized formulations (D1, D5, P1 and P5) at PBS 7.4.

The prepared NSs formulations (D1, D5, P1 and P5) release profiles were shown in Fig. 4. The tested formulations' release profiles displayed a biphasic behavior, with an initial quick release lasting for the first six hours. This first rapid release may have been caused by the drug's adsorption on the NSs surface vesicles, which led to a fast release from NSs^104^ followed by a delayed, slow release for 24 h.

Frequent administration is the main disadvantage of the majority of the traditional, commercially available drug delivery devices. The medication, however, is kept and released gradually over time after being loaded into the Nano sponge. It has been previously reported that hydrophilic cyclodextrin nano sponges are used to adjust the drug release rate, as it facilitates medication absorption over biological barriers and it may help to protect the medication throughout its passage through the stomach^105,106^.

The correlation coefficient (R^2^) values of NSs formulations demonstrated a superior fit to Higuchi's model compared to the zero order and first order kinetic models. This conclusion was drawn from a linear regression analysis of the mathematical models employed to analyze the release data obtained from the NSs formulations. The R^2^ values ranged from 0.8752 to 0.9943. In instances where a high degree of linearity was observed, the Peppas equation was utilized to further investigate the release process of DEX and plant material^107,108^. According to the Peppas theory, if the value of n = 0.43, the drug release process adheres to Fickian diffusion. When 0.43 < n < 0.85, the release process deviates from Fickian diffusion and follows anomalous (non-Fickian) diffusion. A value of n = 0.85 signifies case II transport, while n > 0.85 indicates super-case II transport^107^. The range of values for the release exponent "n" in the NSs formulations varied from 0.213 to 0.452, thereby suggesting a release mechanism controlled by Fickian diffusion^109,110^.

### Pharmacological study

#### In vitro antioxidants activity of *Boswellia carterri* extracts

The effects of the time of interaction of several antioxidants on the suppression of the absorbance of the ABTS^+^ radical cation at 734 nm for the standard reference compounds, trolox and ascorbic acid, are shown in Table 4. The total extract, dichloromethane fraction, ethyl acetate fraction, butanol fraction, and water fraction values are compared with those of the two standards.

**Table 4:** In vitro study of comparative DPPH antioxidant activity of standards with different extracts BC plant.

Sample/DPPH conc µg/ml	Vit C	Trolox	Total	CH_2_CL_2_	EtOAc	BuOH	H2O
30	100 ± 0.00	100.00 ± 0.00	100.00 ± 0.00	–	–	–	–
20	78.8 ± 0.56	80.35 ± 0.52	90.40 ± 0.00	87.40 ± 0.30	89.22 ± 0.00	88.77 ± 0.00	89.32 ± 0.07
15	70.91 ± 0.89	75.58 ± 0.75	90.25 ± 0.00	81.54 ± 0.79	85.38 ± 0.00	85.52 ± 0.00	90.55 ± 0.00
10	70.91 ± 0.94	60.59 ± 0.85	89.07 ± 0.32	67.55 ± 0.23	84.79 ± 0.00	83.31 ± 0.00	82.72 ± 3.85
5	70.91 ± 0.33	37.20 ± 0.42	81.09 ± 0.16	42.84 ± 6.15	81.88 ± 0.09	76.07 ± 0.74	60.46 ± 0.09
2.5	70.91 ± 0.52	30.55 ± 0.96	75.37 ± 0.22	–	70.05 ± 0.06	69.48 ± 0.30	36.88 ± 0.17
1.5	–	–	61.30 ± 0.30	–	69.48 ± 0.30	55.59 ± 0.23	–
1	–	–	50.11 ± 0.23	–	55.59 ± 0.23		–
IC_50_	7.808 ± 0.54	6.316 ± 0.58	0.906 ± 0.15	6.070 ± 1.86	0.532 ± 0.09	0.932 ± 0.21	3.639 ± 0.83

Numerous studies have been conducted on the antioxidant properties of vitamin C, trolox, and natural resin extract compounds. Therefore, the purpose of this study is to determine the antioxidant activity of standard compounds generated from different resins with respect to their ability to scavenge DPPH and ABTS radicals. Hazardous free radicals in humans are stabilized in large part by the DPPH and ABTS radical scavenging tests.

Table 4 presents the effects of the duration of interaction of specific antioxidants on the suppression of the absorbance of the ABTS^**+**^ radical cation at 734 nm for trolox, ascorbic acid, and the standard reference compounds. The values of the two standards are compared with those of total extract, dichloromethane fraction, ethyl acetate fraction, butanol fraction, and water fraction.

The antioxidant activity of natural resin extract substances and Vit C and Trolox has been extensively studied. As a result, this study aims to identify the antioxidant activity of standard substances derived from various resins in terms of their DPPH radical scavenging activity and ABTS radical scavenging activity. The DPPH and ABTS radical scavenging assays play a crucial role in stabilizing harmful free radicals in the human body by providing a redox-functioned proton ion for unstable free radicals. This is accomplished by utilizing the fact that unstable violet DPPH and ABTS free radicals convert into stable yellow DPPH free radicals through the acceptance of a hydrogen ion from antioxidants. In this particular study, the results of the antioxidant activities assessed through the DPPH assay reveal that the BC resin ethyl acetate extract exhibits the highest antioxidant activity in both methods (IC_50_ 0.5325 and 0.5527, respectively). It is followed by the butanol fraction as an agent (IC_50_ 0.932 and 0.6476, respectively) when compared to the other extract samples and standard Vit C and Trolox (Tables 4 and 5) and Supplementary Figure S2.

**Table 5:** In vitro study of comparative ABTS antioxidant activity of standards with different extracts BC plant.

Sample/ABTS conc µg/ml	Vit C	Trolox	Total	CH2CL2	EtOAc	BuOH	H2O
30	100 ± 0.00	100 ± 0.00	–	–	–	100 ± 0.00	100 ± 0.00
20	90.23 ± 0.85	83.55 ± 0.81	90.49 ± 0.13	80.15 ± 0.57	87.81 ± 0.00	90.03 ± 0.00	90.49 ± 0.13
15	83.63 ± 0.86	80.77 ± 1.02	89.75 ± 0.00	67.87 ± 3.19	86.7 ± 0.00	89.29 ± 0.13	89.84 ± 0.13
10	70.03 ± 0.99	73.42 ± 0.85	89.57 ± 0.16	63.07 ± 4.16	85.87 ± 0.00	88.64 ± 0.00	90.12 ± 0.58
5	56.19 ± 0.25	45.21 ± 0.89	83.47 ± 0.16	35.46 ± 1.54	86.06 ± 0.16	87.17 ± 0.16	86.8 ± 0.8
2.5	20.52 ± 0.65	24.2 ± 0.85	75.37 ± 0.23	–	70.05 ± 0.06	75.18 ± 0.06	36.88 ± 0.17
1.5	–	–	61.3 ± 0.04	–	69.48 ± 0.3	69.48 ± 0.3	–
1	–	–	50.11 ± 0.1	–	55.59 ± 0.23	55.59 ± 0.23	–
IC50	5.043 ± 0.60	5.482 ± 0.73	0.887 ± 0.11	7.514 ± 2.36	0.553 ± 0.10	0.647 ± 0.11	2.943 ± 0.30

The two methodologies employed in this section to gauge the antioxidant activity mutually corroborated one another, as all fractions were taken into account. This implies that the examined extracts may possess comparable chemical groups, and their effects can be attributed to these groups^111^. In this regard, the identification and characterization of Cembrane-type diterpenoids^112^, Prenylaromadendrane-type diterpenoids, Triterpenes belonging to tirucallane, ursane, oleanane, and lupane types, as well as polyphenolics, prove instrumental in the development of natural antioxidant substances derived from BC resins^113^. Boswellic acids have been documented for their antioxidant properties owing to their capability to enhance the concentration of reduced glutathione (GSH). This characteristic is also evident in their anti-inflammatory, antitumor, and immunomodulatory effects^114^. The outcomes of the current research on *BC* align with the discoveries of Safayhi et al.^115^, who identified Boswellic acids as specific inhibitors of the 5-lipoxygenase (5-LO) product formation that belong to the non-reducing type. These inhibitors achieve their function by either directly interacting with the 5-LO enzyme or obstructing its translocation.

#### In vivo pharmacological study

The present study, observation of the negative control rats didn’t reveal any abnormal respiratory sign as their nostrils appearances and nasal colours were normal as well as their attitudes towards the examiner and caregiver, these signs were confirmed with the measured biochemical parameters of allergy and inflammation as “ICAM-1, Ilβ4 and LTB_4_”, that were estimated in their sera, and were significantly less than the positive control group (Table 6). On the other hand, signs of severe induced respiratory distress in all the rats that were powdered with talc powder for 4 weeks, and then left untreated for another 4 weeks (positive control group), were manifested by decreased food consumption, avoidance, irritability, increased aggression towards the researcher and the care-giver, hair ruffling, increased nasal discharge, nasal oedema and discolouration, together with significant elevation of ICAM-1, Ilβ4 and LT B4, compared to the negative control group.

**Table 6:** Effects of treatment with *Boswellia carterii* ethyl acetate plant extract, Dexa salt and their nano sponge formulations on inflammatory and allergic mediators of respiratory tract.

Group	Parameter		
	ICAM-1 (pg/ml)	Il*β* 4 (pg/ml)	LTB_4_(pg/ml)
Negative control	351 ± 13.17	1490 ± 8.477	2660 ± 29.39
Positive control (untreated talcosis)	1323 ± 12.94^a^	4604 ± 36.11^a^	3973 ± 19.57^a^
Dexa salt	2652 ± 101.4^ab^	4666 ± 88.82^a^	4401 ± 85.77^a^
DEX NSs (D1) (20 µg)	1501 ± 8.197^abc^	3715 ± 33.05^abc^	3973 ± 60.05^a^
DEX NSs (D5) (20 µg)	400 ± 7.579^bc^	3524 ± 16.12^abc^	5168 ± 229^abc^
*B. carterii* ethyl acetate Plant extract	802 ± 13.56^abc^	2309 ± 4.980^abc^	2706 ± 43.58^bc^
*B. carterii* NSs (P1), (20 µg)	343 ± 9.151^bcd^	2566 ± 22.44^abcd^	2658 ± 25.63^bc^
*B. carterii* NSs (P2), (20 µg)	858 ± 17.61^abc^	2847 ± 17.24^abcd^	2190 ± 93.59^abcd^
DF1 NSs (20 µg)	1295 ± 15.61^acd^	3550 ± 24.36^abcd^	3610 ± 15.38^acd^
DF2 NSs (20 µg)	1303 ± 8.47^acd^	2847 ± 17.24^abcd^	3460 ± 17.86^abcd^

Results are expressed as means ± S.E, N = 5. Comparisons between means were carried out using one way analysis of variance (ANOVA) followed by Tukey Kramer’s multiple comparisons test. p ≤ 0.0001.

^a^Significantly different from Negative control group, ^b^Significantly different from positive control group, ^c^Significantly different from Dexa salt group, ^d^Significantly different from Boswellia extract group.

In the clinical trial done by Villar et al.^116^; it has been reported that there had been no proven therapy for acute respiratory distress, before they started performing their trial on the therapeutic effect of DEX regarding improving the quality of life of patients and reducing their mortality rate. The effect of DEX was still controversial, although, it had some therapeutic action yet, it caused hyperglycemia and the incidence of adverse effects wasn’t significantly different from untreated groups. The controversial effect was clear in our study, as, treatment with conventional DEX salt preparation didn’t improve some of the manifestations of respiratory distress observed in rats as irritability, high aggression, reduced food consumption and avoidance, but it reduced nasal edema and discharge, yet it didn’t improve the biochemical parameters as shown in Table 6, when compared to the positive control group. On the contrary, it significantly elevated the ICAM-1 compared to positive control group. As for the group treated with D1; it reduced nasal edema and discolouration, and has significantly lowered the Il*β*4 levels when compared to positive control group, without affecting the levels of ICAM-1 or LTB_4_, denoting that it exerted only an anti-inflammatory effect without any anti-allergic potential. The best effect of DEX salt treatment was obtained with D5, as it significantly lowered the ICAM-1 and Il*β*4, however it significantly elevated the LTB_4_ levels in sera, which means that it has both anti-inflammatory and to some extent anti-allergic therapeutic potentials on respiratory tract allergies.

Treatment with *Boswellia* extract and its Nano sponge’s formulations (P1 and P2) improved significantly, but with different extents, all manifestations of respiratory distress observed before starting treatment of rats, they also improved significantly the biochemical parameters mentioned in Table 6, when compared to both conventional DEX salt and positive control groups.

Regarding treatment with Drug-free NSs (DF1 and DF2), they showed significant less levels of ICAM-1, when compared to conventional DEX salt group, but didn’t significantly show any difference in levels when compared to the positive control group; yet, it was significantly higher than *Boswellia* extract group level. The levels of Il*β*4 and LTB_4_ in both groups were significantly less than positive control and conventional DEX salt groups, but higher than *Boswellia* extract group (Table 6). This indicates that drug-free nano-sponge have relative anti-allergic and anti-inflammatory effects but not limited to respiratory tract allergy only^117^.

“Intercellular adhesion molecule 1 (ICAM-1)”, is a bispecific antibody that is extensively expressed on the respiratory epithelial cells of allergic patients^118^. ICAM-1 protein increases in airways of asthmatics^119^. Therefore, its level is a strong indicator of the effect of allergen and therapeutics, which target the respiratory system. Based on this fact, the results of the present study suggest that, the best effect obtained was that of *Boswellia* extract in Nano-sponge (P1), as it significantly lowered the ICAM-1 when compared to the positive control group and was near the normal level when compared to negative control group without significant difference, moreover it was significantly less than both conventional DEX salt and *Boswellia* extract levels.

Leukotrienes LTB_4_ is a lipid mediator that participates in the incidence of severe asthma or asthmatic exacerbations via acting as a potent neutrophil chemoattractant and via activation of CD_4_ + T cells, eosinophils and macrophages. That’s why, its blockage is the target for respiratory allergies and asthma control^120^. Additionally, the cytokine “Interleukin 4 (IL*β*-4)” plays a crucial role in induction and differentiation of T- helper cells. One of the major causes of asthma and inflammatory airway diseases are induced via IL*β*-4^121^. It was found in the present study that *Boswellia* extract in Nano-sponge P1 also, lowered significantly both Ilβ4 and LTB_4_ levels compared to both conventional DEX salt and positive control group. The effect of *Boswellia* in this study is in agreement with the findings of Taha et al.^122^; in their study who stated that *Boswellia carterii* resins suppressed cough in rats via blocking leukotriene pathway^122^. Also, Nemat et al.^123^ attributed the neuroprotective effects of *Boswellia* to its anti-leukotriene action thus exhibition of anti-inflammatory and immunomodulatory potentials.

**Figure 5:**
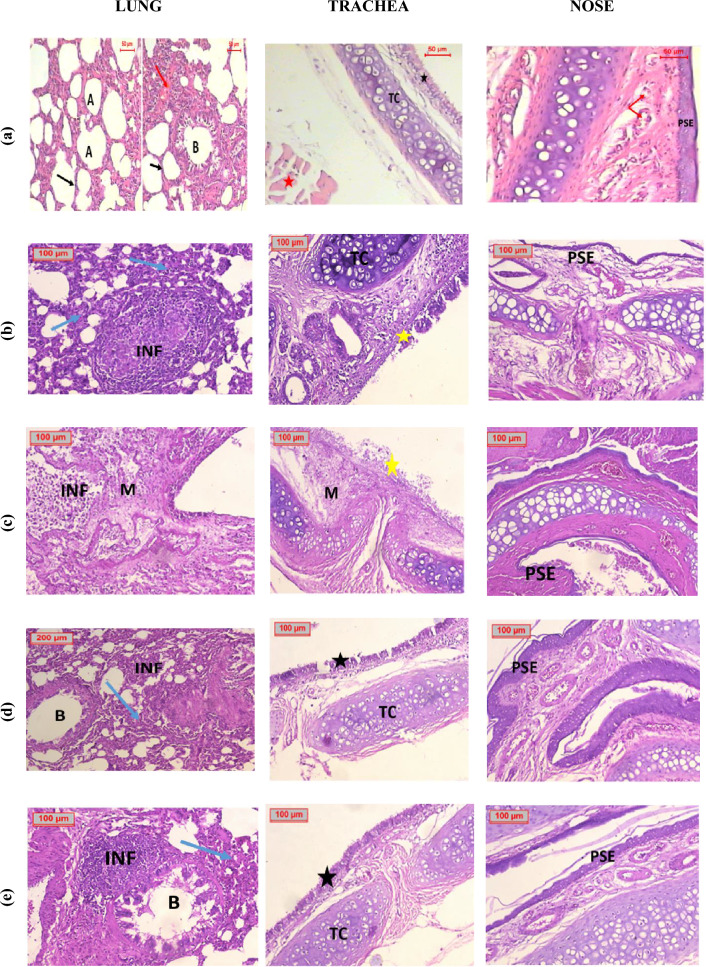

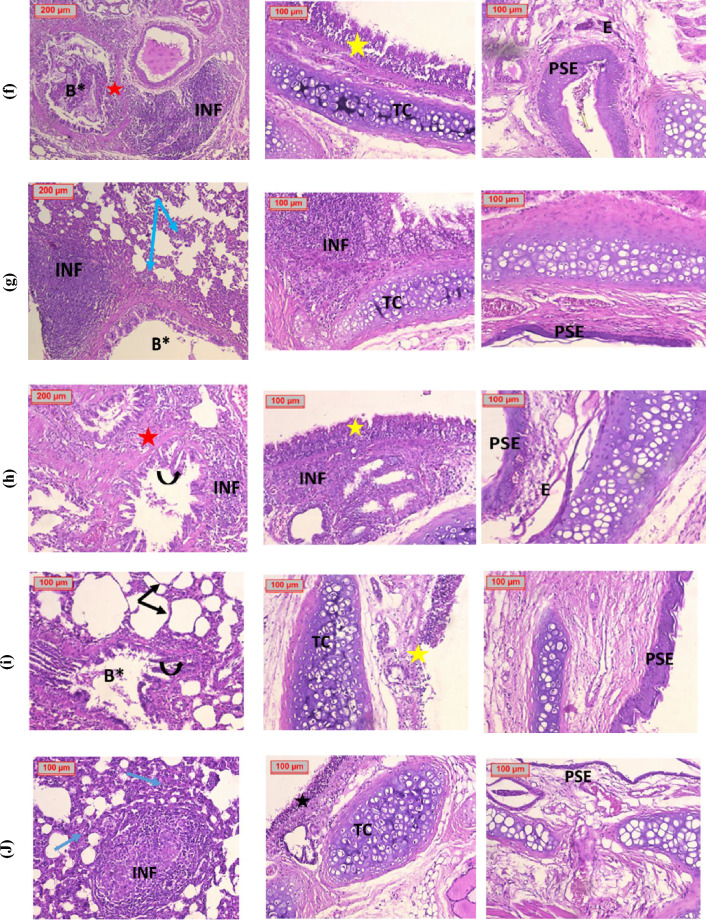
Photomicrographs of lung, tracheal and nasal tissues of (**a**) Negative control, (**b**) Positive control, (**c**) DEX salt, (**d**) DEX salt Nano sponges (D1), (**e**) DEX salt Nano sponges (D5), (**f**) *Boswellia* Extract (**g**) Plant extract Nano sponges (P1), (**h**) Plant extract Nano sponges (P2), (**i**) Drug-free NSs DF1 and (**J**) Drug-free NSs DF2. *Blue arrows*; alveoli with increased thickness wall, *Black arrows*; alveoli with average thickness wall, *Red arrows*; bronchial vessels have normal looking and average thickness, *Dashed red arrows*; congested blood vessels, *A*; Alveoli, *B*; bronchioles, B*; bronchioles filled with mucus, *INF* inflammatory cells infiltrates, *Black stars*; ciliated pseudostratified columnar epithelium, *Yellow stars*; hyperplastic columnar epithelium, *TC* tracheal cartilage, *PSE* pseudostratified epithelium. All photomicrographs were taken at two magnification powers (× 200 and × 400).

*Histopathological examinations* Histopathological examination of the lungs of negative control group showed that they were more like a well-organized sponge consisting of functional respiratory units alveoli, each alveolus shared its wall (inter-alveolar septum) with adjacent alveoli, they were average thickness, the bronchioles were lined by columnar epithelium supported by smooth muscle layer had normal appearance and the bronchial vessels had normal looking and average thickness. The tracheas were lined by ciliate pseudostratified columnar epithelium, resting upon ordinary connective tissue with incomplete rings of hyaline cartilage. The nasal cavities were lined by pseudostratified epithelial mucosa and submucosal regions were filled of blood vessels, mucin and serous secreting gland (Fig. 5a).

On the other hand, histopathological examination of tissues from positive control group (Fig. 5b), showed signs of respiratory distress in the form of marked panting and tachypnea compared to the other groups. Naik and Guruprasad^124^, stated that talc powder causes dryness of the tracheobronchial mucosa and impairs its ciliary function; moreover, the adsorption of the surfactant to the magnesium silicate powder augments the lung injury^114^, this empathizes the pathological findings seen in our study including oedema, inflammation of the bronchial, epithelium, diffuse infiltrates, and lung injury, that consequently led to acute respiratory distress syndrome. The histopathological examination of tissues excised from rats of this group, revealed that lung tissues were massively infiltrated with inflammatory cells infiltrates with germinal formation and destruction of bronchial walls, also the alveolar walls were thickened due to infiltration by inflammatory cells. Examination of the tracheal tissue revealed severe destruction of the tracheal lining with inflammatory cells infiltrate of submucosal tissue, and destruction of tracheal cartilage. Moreover, nasal tissue examination revealed that the nasal cavity was lined by pseudostratified epithelium, mucosa and submucosa region filled of blood vessels, mucin and serous secreting gland^125^.

The biochemical investigations were confirmed by histopathological examination of lung tissues excised from DEX salt treated group (Fig. 5c), as it was found that the lung tissues were mostly destructed by severe infiltration of inflammatory cells with focal areas of mucin, Tracheal tissues of DEX salt treated group, showed that the tracheal lining was destructed and rested upon connective tissue infiltrated by inflammatory cells and focal areas of mucin.

Histopathological examination of lungs of animals treated with D1 and D5 showed infiltration of inflammatory cells with evidence of germinal formation and thickened alveolar walls (Fig. 5d and e). On the other hand, tracheas tissues damage has been improved. Nasal tissue revealed that the nasal cavity was lined by pseudostratified epithelium, mucosa and the submucosa region filled of blood vessels, mucin and serous secreting gland confirming the healing of nasal tissues^126^.

Histopathological examination of lung tissues of *Boswellia* extract treated group (Fig. 5f) revealed that the lung was more likely to destructed by massive inflammatory cells infiltrates which also destructed the muscular walls of the bronchi also totally invade their lumens. Also, the group treated with P1 showed destructive bronchial architecture by inflammatory cells, and some had focal formation, the alveolar walls were infiltrated by inflammatory cells (Fig. 5g), the same picture was presented in the group treated with P5 which showed indentation of bronchial lining with evidence of intra-bronchial mucous with destruction of bronchial and alveolar walls due to inflammatory cells infiltration (Fig. 5h).

Examination of tracheal tissues of *Boswellia* extract group and the group treated with P2 revealed hyperplastic columnar epithelium with inflammatory cells infiltrates. In addition, the group treated with P1 showed massively destructed tracheal lining as it was infiltrated by inflammatory cells. Nasal tissue examination of groups treated with *Boswellia* extract and with P2 showed disturbed mucosal region by edema and inflammatory cell infiltrate, this picture is improved in the group treated with P1 as there are minimal inflammatory cell infiltrate with congested dilated blood vessels within mucosa and submucosal region.

Histopathological examination of lung tissues of the group treated with Drug free Nano-sponge DF1 showed massively inflammatory cells infiltrates with destruction of bronchial walls; while, the group treated with Drug free Nano-sponge DF2 showed more infiltration of inflammatory cells, with germinal formation also the alveolar walls were thickened due to infiltration by inflammatory cells. On the other hand, examination of tracheal tissue of the group treated with DF1, revealed more destruction of the tracheal lining with inflammatory cells infiltrate of submucosal tissue. The previous pathological picture showed improvement with minimal inflammatory cells infiltrate in the group treated with DF2. Nasal tissue examination of both groups showed that the nasal cavity was lined by pseudostratified epithelium , mucosa and submucosa region filled of blood vessels, mucin and serous secreting gland (Fig. 5i and J).

## Conclusion

The successful extraction and characterization of the ethyl acetate plant extract from *Boswellia carterii* were effectively accomplished. The concentration of compound groups in BC-Resins, particularly Cembrane-type diterpenoids, is notably higher compared to other groups listed in Table 1. These compounds have demonstrated significant capabilities in inhibiting the production of TNF-*α* and IL-6, along with the expression of iNOS, COX-2, and *p*-TNF-κB. Furthermore, the compound exhibits strong inhibitory effects against the inflammatory enzyme 5-lipoxygenase. Nano sponges loaded with either *Boswellia carterii* ethyl acetate plant extract or dexamethasone salt exhibited a majority of the favorable attributes required for an appropriate dosage form. The development of small-sized nano sponges has been successfully executed. The release profiles achieved a sustained drug release for a duration of 24 h. Moreover, these nano sponge formulations have demonstrated a considerable capability to prolong drug release time, leading to potential advantages such as reduced drug administration frequency, lower medication doses, and prevention of systemic adverse effects. The anti-inflammatory properties *of Boswellia carterii* ethyl acetate plant extract, dexamethasone salt, and their nano formulations (D1, D5, P1, and P2) in treating respiratory allergies were assessed. Examination of histopathological samples and measurement of intracellular adhesion molecule-1 (ICAM-1), Leukotriene B4 (LTB4), and Interleukin *β* 4 (IL*β* 4) levels indicated a significant reduction in inflammatory biomarkers in treated rats, along with improved histopathological characteristics compared to the positive control group. The ethyl acetate extract of *Boswellia* species and its nano sponge formulation P1 exhibited promising therapeutic effects on both upper and lower respiratory ailments. The therapeutic impact of the *Boswellia* ethyl acetate extract formulation P1 appears to stem from a synergistic interaction between the extract and DF1, achieved by dual inhibition of the ICAM-1 and LTB4 pathways, effectively counteracting the allergic and inflammatory effects induced by talc powder.

### Supplementary Information

Supplementary Tables.

## Data Availability

The datasets used during the current study available from the corresponding author on reasonable request.

## Abbreviations

BC: *Boswellia carterii*
CID: Collision-induced dissociation
HESI: Heated electrospray ionization
C + ve: Positive control group
DPPH: 2,2-Diphenyl-1-picrylhydrazyl
DEX: Dexamethasone salt
ICAM-1: Intracellular adhesion molecule-1
Il*β* 4: Interleukin*β* 4
LTB_4_: Leukotriene B4
CD: Cyclodextrin
CA: Citric acid
ABTS: 2,2'-Azino-bis(3-ethylbenzothiazoline-6-sulfonic acid)
PMDA: Pyromellitic dianhydride
CDI: 1, 1′-Carbonyldiimidazole
DPC: Diphenyl carbonate
LCMSMS: Liquid chromatography–mass spectrometry
HP*β*-CD: Hydroxypropyl *β*-cyclodextrin
abf: Common output format
RPM: Rotations per minute
IAEC: Institutional animal ethical committee
UPLC: Ultra-performance liquid chromatography
EPI-*β* -CD: Epichlorohydrin-*β*-cyclodextrin
BDE: 1,4-Butanediol diglycidyl ether
EtOAc: Ethyl acetate
RI: Retention indices
