# Supplementary material for: Appraisal terpenoids rich Boswellia carterri ethyl acetate extract in binary cyclodextrin oligomer nano complex for improving respiratory distress
Source: Sci Rep. 2024 Jul 22;14:16779. doi: 10.1038/s41598-024-66297-2 (PMC11263383; doi:10.1038/s41598-024-66297-2)
Supplement: Supplementary file 1 — Supplementary Tables. [file 41598_2024_66297_MOESM1_ESM.docx]

**QC**

**MeOH**

**EtOAc**

**Fig. (S1). profiles of listed compounds in the table 3 by LC-MSMS.**


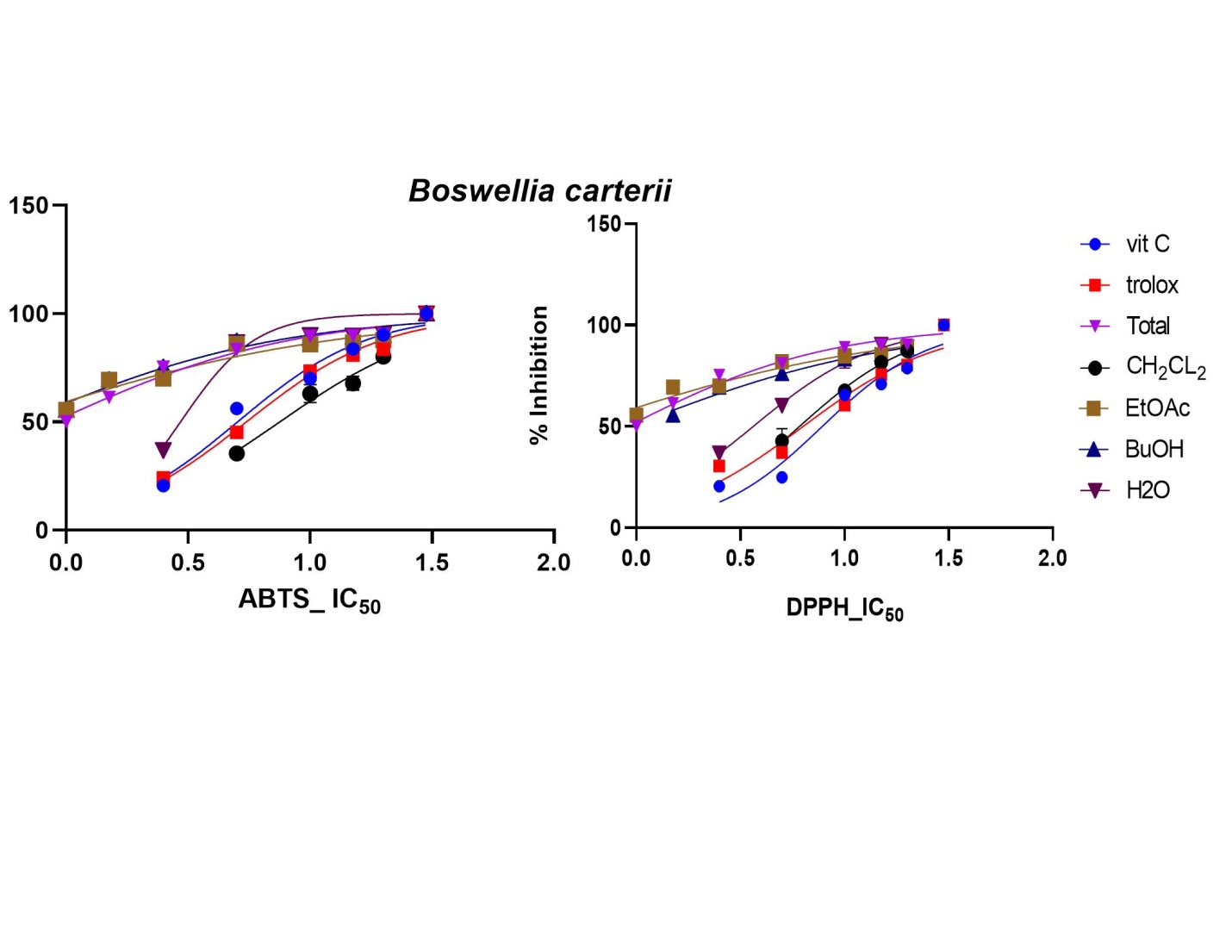
 Fig. S2. *In vitro* study of IC 50 DPPH and ABTS^+^ antioxidant activity for different extracts BC plant as (ug/ml).
